# Effects of electroacupuncture on neurological outcomes and brain–gut axis-associated changes in experimental ischemic stroke models: a systematic review and meta-analysis

**DOI:** 10.3389/fnins.2026.1893796

**Published:** 2026-08-25

**Authors:** Yizhou Chen, Tao Zhu, Ming Li, Tianying Tan, Jin Cui

**Affiliations:** 1 Guizhou University of Traditional Chinese Medicine, Guiyang, China; 2 The First Affiliated Hospital of Guizhou University of Traditional Chinese Medicine, Guiyang, China

**Keywords:** brain–gut axis, electroacupuncture, gut microbiota, intestinal barrier, stroke

## Abstract

**Background:**

Ischemic stroke is a leading cause of death and disability worldwide and is frequently accompanied by brain–gut axis disturbances, including intestinal barrier dysfunction, gut microbiota dysbiosis, and immune–inflammatory imbalance. Although electroacupuncture (EA) has demonstrated neuroprotective effects in experimental stroke, its effects on brain–gut axis-related outcomes have not been systematically evaluated.

**Objective:**

To evaluate the effects of EA on neurological outcomes and brain–gut axis-related changes in animal models of ischemic stroke.

**Methods:**

This systematic review and meta-analysis was conducted in accordance with the PRISMA 2020 statement and registered in PROSPERO (CRD420261354722). Nine databases were searched from inception to 1 May 2026. Controlled animal studies comparing EA with non-EA controls in ischemic stroke models were included. Random-effects meta-analyses were performed using Hedges’ *g* standardized mean differences (SMDs) with 95% confidence intervals (CIs). Risk of bias was assessed using the SYRCLE tool, and the certainty of evidence was evaluated using an adapted GRADE framework for preclinical animal studies.

**Results:**

Thirteen reports representing 12 independent studies were included. EA was associated with improved neurological function (11 studies; SMD = −2.49, 95% CI: −3.50 to −1.47; *I*^2^ = 60.5%) and reduced infarct volume (9 studies; SMD = −3.08, 95% CI: −4.22 to −1.95; *I*^2^ = 56.2%). EA was also associated with increased ZO-1, occludin, and intestinal IL-10 levels and reduced D-lactate, lipopolysaccharide, intestinal IL-1β, and intestinal TNF-α levels. Qualitative synthesis suggested EA-associated changes in gut microbial diversity and taxonomic composition, although their functional significance remains uncertain. The certainty of evidence ranged from moderate to very low, and several brain–gut axis-related outcomes were supported by only a small number of studies.

**Conclusion:**

EA-related neuroprotection in ischemic stroke may be partially associated with modulation of brain–gut axis-related processes; however, the available evidence does not establish these processes as the primary mediator. Further rigorously designed preclinical studies are needed to strengthen causal inference and facilitate clinical translation.

**Systematic review registration:**

https://www.crd.york.ac.uk/PROSPERO/view/CRD420261354722, Identifier: CRD420261354722.

## 1 Introduction

Stroke is an acute cerebrovascular disease and the second leading cause of death worldwide, accounting for approximately 5.5 million deaths annually. It is characterized by high incidence, disability, and mortality (Zhang et al., 2026). Stroke is broadly classified into ischemic and hemorrhagic subtypes, of which ischemic stroke is the most common, accounting for approximately 84% of cases worldwide (Ma et al., 2021). Despite continued advances in reperfusion therapy, many patients still experience varying degrees of neurological dysfunction after the acute phase, underscoring the need to explore novel adjunctive interventions and their underlying mechanisms (Furie and Kelly, 2026).

Ischemic stroke is initiated by cerebral arterial occlusion and the resulting reduction in cerebral blood flow, which trigger energy failure, excitotoxicity, oxidative stress, and neuronal injury. Brain–gut axis dysfunction is therefore unlikely to represent the initiating cause of ischemic stroke. Rather, it may emerge after the primary cerebral insult and contribute to secondary injury, systemic responses, complications, and recovery. Within this context, accumulating evidence indicates that ischemic stroke is frequently accompanied by gut microbiota dysbiosis, impaired intestinal barrier function, and immune-inflammatory imbalance, which may in turn influence the subsequent evolution and prognosis of brain injury (Guo H. et al., 2025; Ma et al., 2024; Zhang et al., 2024). Post-stroke intestinal alterations may extend beyond microbial composition to involve remodeling of the intestinal microenvironment, immune homeostasis, and metabolic networks, while microbial metabolites and immunoregulatory processes may provide links between peripheral responses and secondary brain injury (Wang T. et al., 2023). Clinical studies have also associated gut microbiota composition and plasma metabolite profiles with stroke-related complications and outcomes, although these associations do not establish a role in stroke initiation (Lin et al., 2025; Wang et al., 2024; Yan et al., 2025c).

Electroacupuncture (EA), an important form of acupuncture therapy, has been widely applied in basic and clinical studies of ischemic stroke because its stimulation parameters are relatively controllable and reproducible. In rodent experiments, EA generally involves inserting acupuncture needles at predefined anatomical sites and connecting them to an electrical pulse generator. The procedure is characterized by the selected acupoints, electrode configuration, stimulation frequency and waveform, current intensity, session duration, treatment frequency, and treatment course. From a neurophysiological perspective, EA acts initially as an external somatosensory stimulus that engages peripheral sensory afferents and central/autonomic regulatory networks rather than directly targeting intestinal microorganisms (Li Y. W. et al., 2022). These neural signals may subsequently influence neuroimmune and systemic biological responses. Accordingly, intestinal and microbial alterations observed after EA may represent downstream, parallel, or feedback responses rather than its initial direct targets.

However, existing preclinical evidence on EA for ischemic stroke remains fragmented across domains such as neurological function, cerebral infarction, intestinal barrier integrity, intestinal inflammation/immunity, and gut microbiota, with little systematic integration from a brain–gut axis perspective. Therefore, this study conducted a systematic review and meta-analysis to comprehensively evaluate the effects of EA on neurological function and brain–gut axis-related outcomes in animal models of ischemic stroke, with the aim of providing an integrated synthesis of the current preclinical evidence and clarifying the potential associations between EA-related neuroprotection and brain–gut axis-related changes.

## 2 Method

### 2.1 Reporting guideline and registration

This systematic review and meta-analysis was conducted in accordance with the Preferred Reporting Items for Systematic Reviews and Meta-Analyses 2020 (PRISMA 2020) statement. The PRISMA 2020 checklist is provided in Supplementary File 1. The protocol was prospectively registered in the International Prospective Register of Systematic Reviews (PROSPERO; registration no. CRD420261354722).

### 2.2 Search strategy

Two reviewers independently conducted a comprehensive literature search in PubMed, Embase, Web of Science Core Collection, the Cochrane Library, Scopus, China National Knowledge Infrastructure (CNKI), VIP Database, Wanfang Database, and China Biology Medicine disc (CBM) from database inception to 1 May 2026. The search strategy combined subject headings and free-text terms related to ischemic stroke, EA, and brain–gut axis-related outcomes, including gut microbiota, intestinal barrier dysfunction, intestinal inflammation, and immune-related markers. The search strategy was adapted to the indexing system and search interface of each database. In addition, the reference lists of relevant reviews and all included studies were manually screened to identify additional eligible records. Detailed search strategies for all databases are provided in Supplementary File 2.

### 2.3 Eligibility criteria

Studies were selected according to the PICOS framework, which includes participants, interventions, comparators, outcomes, and study design. Eligible studies met the following criteria: (1) used animal models of ischemic stroke; (2) evaluated EA as the primary intervention, including either pretreatment or post-modelling treatment; (3) included a non-EA control group; (4) reported at least one brain–gut axis-related outcome, with or without neurological or infarct-related outcomes; and (5) were controlled animal experiments.

### 2.4 Data extraction

Two reviewers independently extracted data from the included studies using a predefined, standardized form. The extracted information included publication details (first author and year), animal characteristics (species and sex), model characteristics, details of the EA intervention (acupoints, frequency, intensity, duration, treatment timing, and treatment course), control conditions, and reported outcomes. The outcomes extracted for this review included neurological function, infarct size, and brain–gut axis-related outcomes, including intestinal barrier-related markers, inflammatory factors, microbiota-related measures, and exploratory gut-related indicators.

For the meta-analysis, numerical data for each outcome were extracted as sample size, mean, and standard deviation for the EA and control groups. When outcomes were reported only graphically, the corresponding values were estimated from the figures. For studies with potentially overlapping datasets or companion reports, the original experimental source was carefully assessed, and overlapping reports were treated as a single study source when appropriate. Discrepancies in data extraction were resolved through discussion between the two reviewers, with arbitration by a third reviewer if necessary.

### 2.5 Risk of bias and study quality assessment

Two reviewers independently assessed the methodological quality and risk of bias of the included studies using the SYRCLE risk of bias tool, which was specifically developed for animal intervention studies (Hooijmans et al., 2014). The assessment covered the following domains: sequence generation, baseline characteristic similarity, allocation concealment, random housing, blinding of caregivers and investigators, random outcome assessment, blinding of outcome assessment, completeness of outcome data, selective outcome reporting, and other potential sources of bias. Each item was rated as having a low, high, or unclear risk of bias. Disagreements were resolved through discussion, with arbitration by a third reviewer when necessary. Risk-of-bias summary plots were generated using the robvis tool.

### 2.6 Certainty of evidence assessment

Two reviewers independently assessed the certainty of evidence for each quantitatively synthesized outcome using an adapted Grading of Recommendations Assessment, Development and Evaluation (GRADE) framework for preclinical animal studies (Hooijmans et al., 2018; Wei et al., 2016). Randomized controlled animal studies were initially considered high-certainty evidence and were downgraded by one or two levels for serious or very serious concerns regarding risk of bias, inconsistency, indirectness, imprecision, or publication bias. Risk-of-bias judgments were made at the outcome level by considering the methodological characteristics of the studies contributing to each pooled estimate, rather than automatically downgrading for isolated unclear ratings. Inconsistency was assessed according to the direction and magnitude of effects, confidence-interval overlap, statistical heterogeneity, and sensitivity analyses. Imprecision was evaluated by considering the total number of animals, the width of the 95% confidence interval, whether the interval crossed the null effect, and the robustness of the pooled estimate. Publication bias was formally evaluated when at least 10 studies were available; for outcomes with fewer than 10 studies, publication bias was considered not formally assessable and was not downgraded solely for this reason. Certainty was classified as high, moderate, low, or very low. Disagreements were resolved through discussion or consultation with a third reviewer.

### 2.7 Statistical analysis

Statistical analyses were performed using R software (version 4.5.2). Random-effects meta-analyses were conducted for all quantitative syntheses. Continuous outcomes were summarized as standardized mean differences (SMDs) with 95% confidence intervals (CIs) using Hedges’ *g*, as similar outcomes were frequently measured using different scales, units, or laboratory methods across studies. Outcome directions were aligned before pooling so that beneficial effects of EA were presented consistently. Where multiple time points or overlapping datasets were reported, the most clinically relevant or non-duplicated dataset was preferentially included in the quantitative synthesis.

Between-study heterogeneity was assessed using Cochran’s *Q* test and quantified with the *I*^2^ statistic. An *I*^2^ value >50% was considered indicative of substantial heterogeneity. Leave-one-out sensitivity analyses were conducted to assess the robustness of the pooled estimates. Exploratory subgroup analyses were performed according to intervention timing when relevant. Funnel plots were generated for visual exploration of potential small-study effects. Egger’s regression test was performed only when at least 10 independent study sources were available for a given outcome; for outcomes with fewer than 10 study sources, funnel plots were interpreted descriptively and no formal test for funnel-plot asymmetry was conducted. Outcomes that could not be appropriately pooled because of limited numbers of studies, inconsistent definitions, or a lack of extractable quantitative data were synthesized qualitatively. A two-sided *p* value <0.05 was considered statistically significant.

## 3 Results

### 3.1 Search result

**Figure 1:**
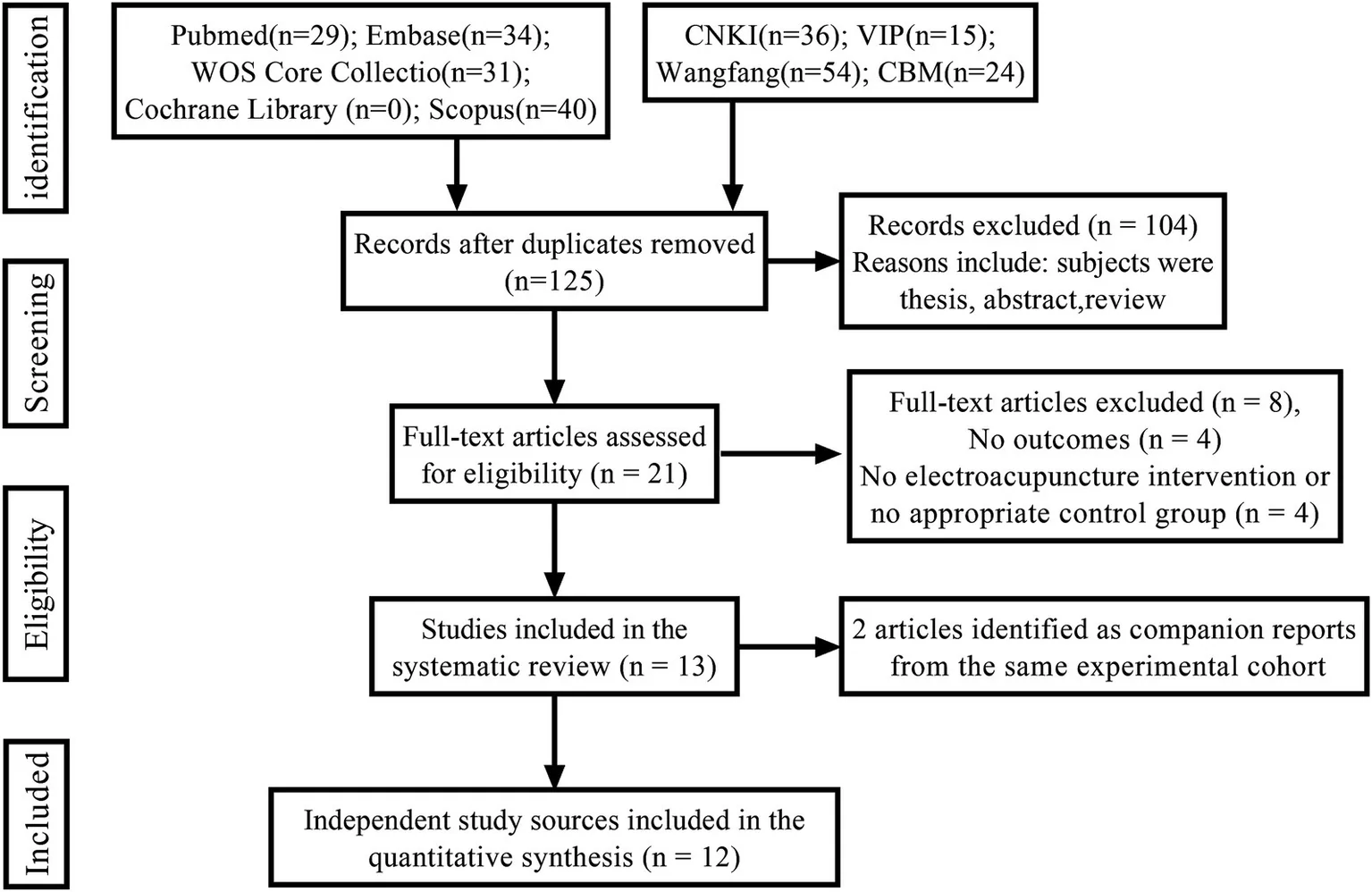
PRISMA flow diagram of study selection.

A total of 263 records were retrieved from PubMed (*n* = 29), Embase (*n* = 34), Web of Science Core Collection (*n* = 31), the Cochrane Library (*n* = 0), Scopus (*n* = 40), CNKI (*n* = 36), VIP (*n* = 15), Wanfang Database (*n* = 54), and CBM (*n* = 24). After duplicate removal, 125 records remained for screening. During the screening stage, 104 records were excluded, including theses, conference abstracts, and reviews. The remaining 21 records were assessed in full text for eligibility. At the full-text assessment stage, 8 articles were excluded: 4 did not report relevant outcomes, and 4 neither evaluated EA nor included an appropriate control group. Ultimately, 13 studies were included in the systematic review. Among these, two articles were identified as companion reports derived from the same experimental cohort and were therefore treated as a single study source in the quantitative synthesis (Yan et al., 2025a,b). Consequently, 12 independent study sources were included in the meta-analysis (Chen Y. et al., 2025; Chen Y. L. et al., 2025; Ke et al., 2022; Li S. et al., 2022; Wang Y. L. et al., 2023; Wu Y. et al., 2026; Yan et al., 2025b; Ye et al., 2025; Zhang A. et al., 2025; Zhang et al., 2023; Zhang Y. et al., 2025; Zhao Q. K. et al., 2026). The detailed study selection process is shown in Figure 1.

### 3.2 Study characteristics

A total of 13 studies were ultimately included, of which 12 independent study sources were included in the quantitative synthesis. The included studies were published between 2022 and 2026. Most used male Sprague–Dawley rats, whereas a few used C57BL/6 mice. Transient middle cerebral artery occlusion (tMCAO) was the predominant ischemic stroke model, with ischemia durations ranging from 60 to 120 min; one study used a permanent MCAO model.

Across studies, EA interventions varied in initiation timing, acupoint selection, stimulation parameters, session duration, and treatment course, and included both pretreatment and post-modelling treatment. Pretreatment generally began 3–14 days before MCAO, whereas post-modelling treatment was initiated either immediately after MCAO or 24 h later. The most commonly used acupoints were GV20 and ST36, and some studies additionally used GV14, GV24, LI11, ST25, or ST37. Reported stimulation frequencies mainly included 2/15 Hz, 2 Hz, 10 Hz, 1/20 Hz, and 2/5 Hz, and stimulation intensity ranged from 0.5 to 2 mA. Treatment sessions generally lasted 20–30 min, and treatment courses ranged from 1 to 14 days. The control groups mainly consisted of ischemic model groups that did not receive EA. The main outcomes included neurological function, infarct volume, intestinal barrier-related markers, inflammatory mediators, and gut microbiota-related measures. Detailed characteristics of the included studies are presented in Table 1.

**Table 1:** Characteristics of the included studies.

Study	Species (gender)	Model method (ischemic time)	Intervention start time	Acupoints	Frequency	Intensity	Waveform	Duration (min)	Total treatment courses	Control	Main outcomes
Zhao Q. K. et al. (2026)	SD (M)	tMCAO (120 min)	Immediately after MCAO	ST36	10 Hz	0.5 mA	undescribed	20	Once daily for 7 days (8 sessions)	tMCAO group	Neurological function; infarct volume; gut barrier markers; inflammatory mediators; gut microbiota
Ye et al. (2025)	SD (M)	tMCAO (120 min)	14 d before MCAO	GV20, ST36	2/15 Hz	1 mA	undescribed	30	6 times per week for 2 week (12 sessions)	tMCAO group	Neurological function; gut barrier markers; inflammatory mediators; gut microbiota
Chen Y. et al. (2025)	SD (M)	tMCAO (120 min)	Immediately after MCAO	GV20	2/15 Hz	1 mA	undescribed	30	Once daily for 3 days (4 sessions)	tMCAO group	Neurological function; infarct volume; inflammatory mediators; gut microbiota
Ke et al. (2022)	SD (M)	tMCAO (90 min)	24 h after MCAO	LI11 and ST36	1/20 Hz	undescribed	undescribed	30	Once daily for 14 days (14 sessions)	tMCAO group	Neurological function; infarct volume; gut microbiota
Li S. et al. (2022)	C57BL/6 (M)	tMCAO (90 min)	5 d before MCAO	GV20, GV14, ST36	2/15 Hz	2 mA	undescribed	30	Once daily for 10 days (10 sessions)	tMCAO group	Neurological function; infarct volume; gut microbiota
Zhang et al. (2023)	C57BL/6 (M)	tMCAO (60 min)	Immediately after MCAO	GV20, ST36	2 Hz	1 mA	undescribed	30	Once daily for 3 days (4 sessions)	tMCAO group	Neurological function; inflammatory mediators; gut microbiota
Chen Y. L. et al. (2025)	SD (M)	tMCAO (120 min)	Immediately after MCAO	GV20	2 Hz	1 mA	undescribed	30	Once daily for 3 days (4 sessions)	tMCAO group	Neurological function; infarct volume; inflammatory mediators; gut microbiota
Wang Y. L. et al. (2023)	SD (M)	tMCAO (120 min)	Immediately after MCAO	GV20	2/15 Hz	1 mA	undescribed	20	Once daily for 1 days (2 sessions)	tMCAO group	Neurological function; infarct volume; gut barrier markers; inflammatory mediators
Zhang A. et al. (2025)	SD (M)	tMCAO (120 min)	3 d before MCAO	GV20, ST36	2/15 Hz	1 mA	Disperse wave	30	Once daily for 3 days (3 sessions)	tMCAO group	Neurological function; infarct volume; inflammatory mediators; gut microbiota
Zhang Y. et al. (2025)	SD (M)	tMCAO (120 min)	24 h after MCAO	GV20, GV24	1/20 Hz	2 mA	Disperse wave	30	Once daily for 7 days (7 sessions)	tMCAO group	Neurological function; inflammatory mediators; gut microbiota
Yan et al. (2025b)	SD (M)	tMCAO (120 min)	14 d before MCAO	GV20, ST36	2/15 Hz	1 mA	Disperse wave	30	6 times per week for 2 weeks (12 sessions)	tMCAO group	Neurological function; Infarct volume; inflammatory mediators; gut microbiota
Wu Y. et al. (2026)	SD (M)	pMCAO	24 h after MCAO	GV20, GV14, ST25, ST37	2/5 Hz	2 mA	Disperse wave	20	Once daily for 14 days (14 sessions)	pMCAOgroup	Infarct volume; gut barrier markers; inflammatory mediators

### 3.3 Risk of bias assessment

**Figure 2:**
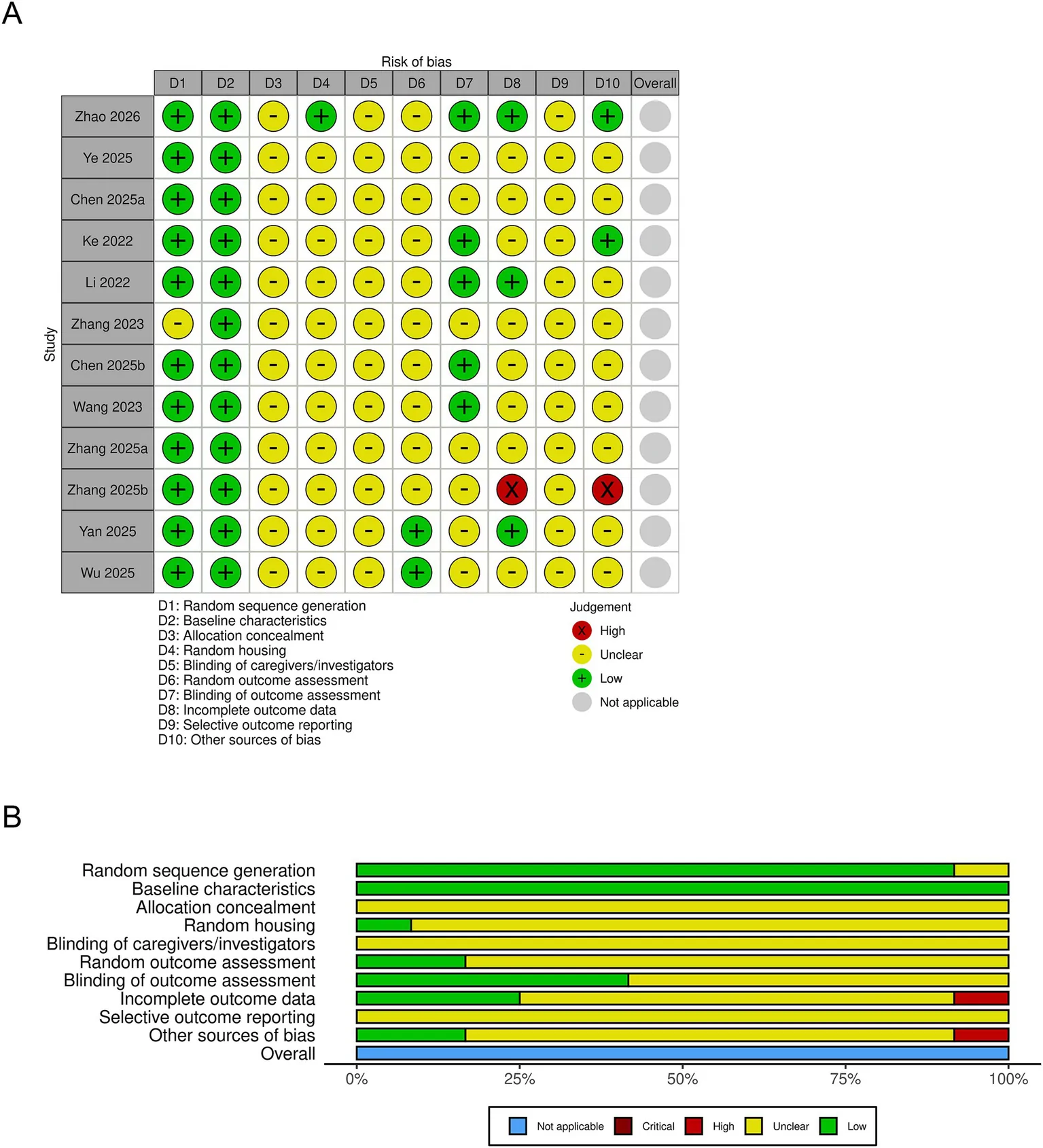
SYRCLE risk-of-bias assessment of the included studies **(A)** Traffic light plot; **(B)** Summary plot.

The results of the risk-of-bias assessment are shown in Figure 2. Overall, the methodological quality of the included studies was moderate, and most domains were judged to be at low or unclear risk of bias. All studies were rated as low risk for baseline characteristic balance, and most were also judged to be at low risk for random sequence generation. In contrast, allocation concealment, blinding of personnel, and selective reporting were mostly judged as unclear because of insufficient methodological reporting. Random housing and random outcome assessment were reported in only a few studies and were therefore mostly judged as unclear. Blinding of outcome assessment was reported in some studies, but overall reporting remained insufficient. Regarding completeness of outcome data, most studies were judged to be at low or unclear risk, although one study was rated as high risk. Similarly, one study was judged to be at high risk for other potential sources of bias.

### 3.4 Primary outcome analyses

#### 3.4.1 Effect of EA on neurological function

**Figure 3:**
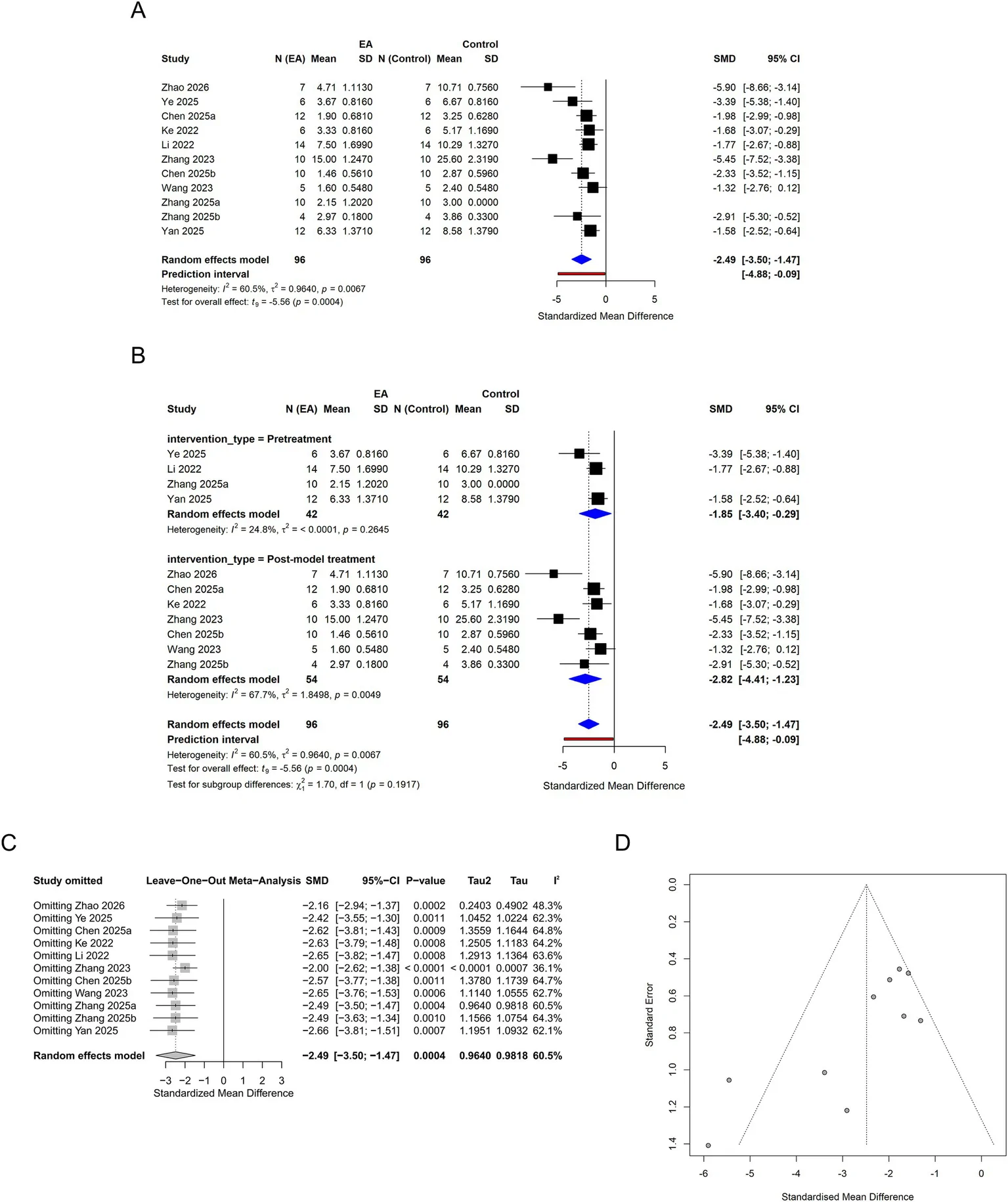
Meta-analysis of the effects of electroacupuncture on neurological function in animal models of ischemic stroke. **(A)** Forest plot of the pooled effect of electroacupuncture on neurological function. **(B)** Subgroup analysis according to intervention timing (pretreatment vs. post-model treatment). **(C)** Leave-one-out sensitivity analysis for neurological function. **(D)** Funnel plot for neurological function.

A total of 11 independent study sources were included. Meta-analysis showed that EA significantly improved neurological function in animal models of ischemic stroke (SMD = −2.490, 95% CI: −3.500 to −1.470, *p* < 0.001), although between-study heterogeneity was high (*I*^2^ = 60.5%, *p* = 0.007) (Figure 3A). Stratified analysis by intervention timing showed beneficial effects of EA in both the pretreatment subgroup (SMD = −1.850, 95% CI: −3.400 to −0.290) and the post-modelling treatment subgroup (SMD = −2.820, 95% CI: −4.410 to −1.230), with no statistically significant difference between subgroups (*p* = 0.192) (Figure 3B). Sensitivity analyses showed that the pooled effect estimate did not change materially after sequential exclusion of individual studies (Figure 3C). Heterogeneity decreased after exclusion of Zhang et al. (2023). The funnel plot showed some asymmetry (Figure 3D), and Egger’s regression test yielded *p* = 0.008.

#### 3.4.2 Effect of EA on infarct volume

**Figure 4:**
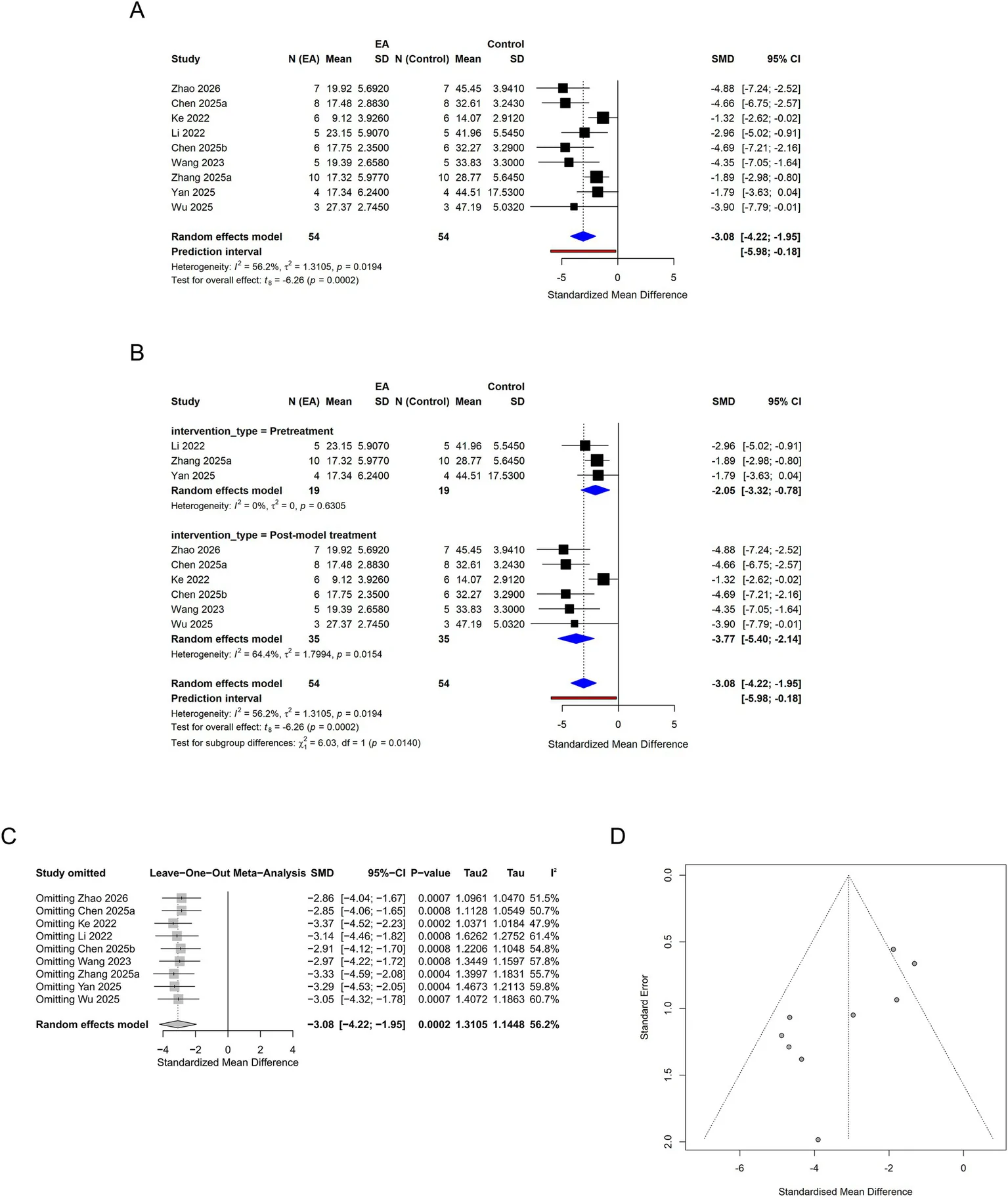
Meta-analysis of the effects of electroacupuncture on infarct volume in animal models of ischemic stroke. **(A)** Forest plot of the pooled effect of electroacupuncture on infarct volume. **(B)** Subgroup analysis according to intervention timing (pretreatment vs. post-model treatment). **(C)** Leave-one-out sensitivity analysis for infarct volume. **(D)** Funnel plot for infarct volume.

A total of 9 independent study sources were included. Meta-analysis showed that EA significantly reduced infarct volume in animal models of ischemic stroke compared with the non-EA control group (SMD = −3.080, 95% CI: −4.220 to −1.950, *p* < 0.001), although moderate between-study heterogeneity was observed (*I*^2^ = 56.2%, *p* = 0.019) (Figure 4A). Stratified analysis by intervention timing showed a pooled effect estimate of SMD = −2.050 (95% CI: −3.320 to −0.780) in the pretreatment subgroup and SMD = −3.770 (95% CI: −5.400 to −2.140) in the post-modelling treatment subgroup, with a statistically significant difference between subgroups (*p* = 0.014) (Figure 4B). Sensitivity analyses showed that the pooled effect estimate did not change materially after sequential exclusion of individual studies (Figure 4C). Because fewer than 10 studies were available for this outcome, only a funnel plot was generated for visual inspection, and no formal publication bias test was performed (Figure 4D).

### 3.5 Brain–gut axis-related outcomes

#### 3.5.1 Gut barrier-related markers

**Figure 5:**
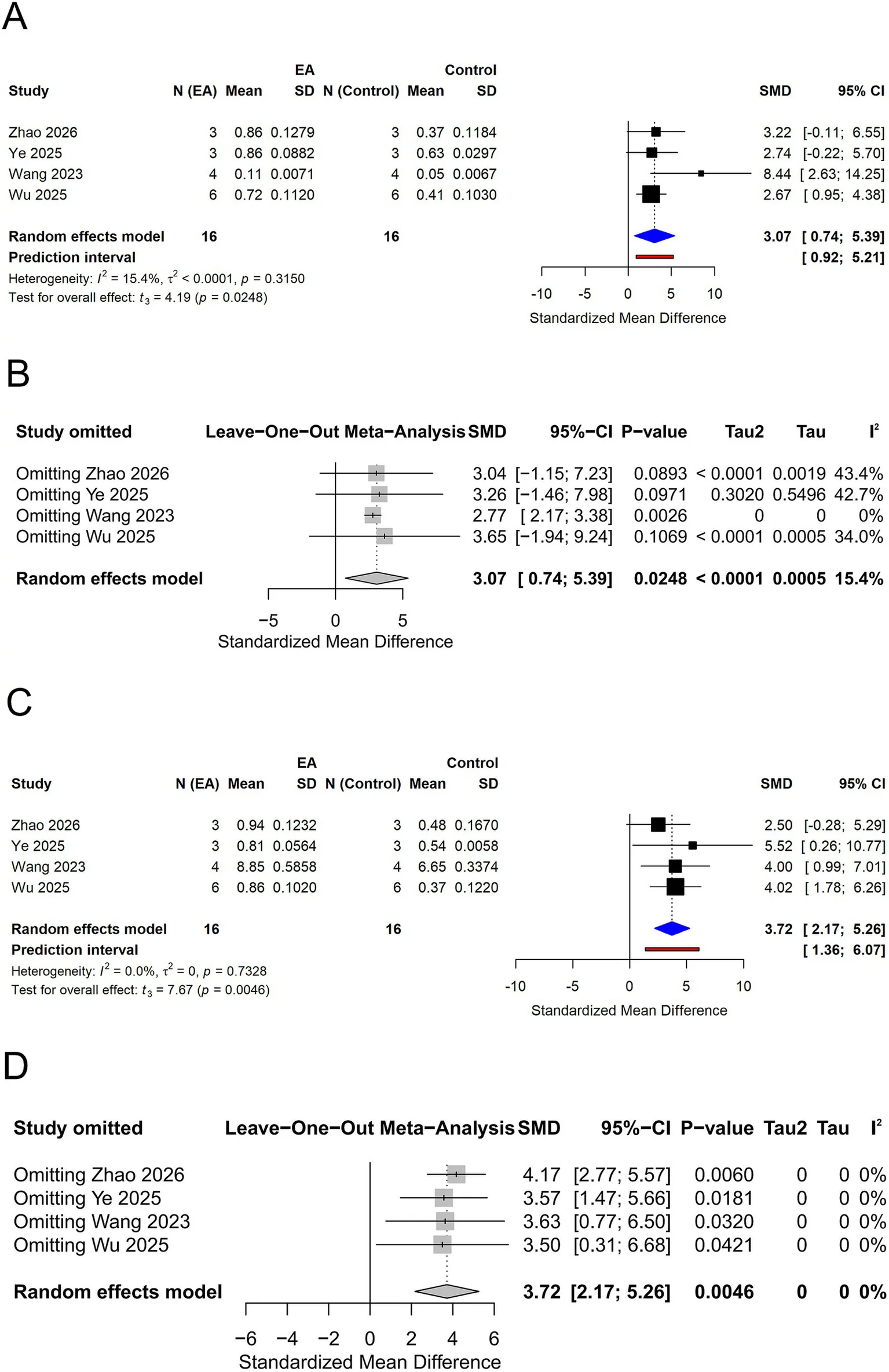
Meta-analysis of the effects of electroacupuncture on intestinal tight junction-related markers. **(A)** Forest plot of the pooled effect of electroacupuncture on ZO-1. **(B)** Leave-one-out sensitivity analysis for ZO-1. **(C)** Forest plot of the pooled effect of electroacupuncture on occludin (Ocln). **(D)** Leave-one-out sensitivity analysis for occludin.

A total of five gut barrier-related markers were quantitatively synthesized, including the intestinal tight junction proteins ZO-1 and Ocln, as well as circulating indicators of barrier injury, namely DAO, D-lactate, and LPS. Meta-analysis showed that EA significantly increased ZO-1 levels (4 studies, SMD = 3.070, 95% CI: 0.740 to 5.390, *p* = 0.025; *I*^2^ = 15.4%) (Figure 5A) and Ocln levels (4 studies, SMD = 3.720, 95% CI: 2.170 to 5.260, *p* = 0.005; *I*^2^ = 0.0%) (Figure 5C). Sensitivity analyses showed that the pooled effect for Ocln remained stable after sequential exclusion of individual studies (Figure 5D), whereas that for ZO-1 was less stable (Figure 5B).

**Figure 6:**
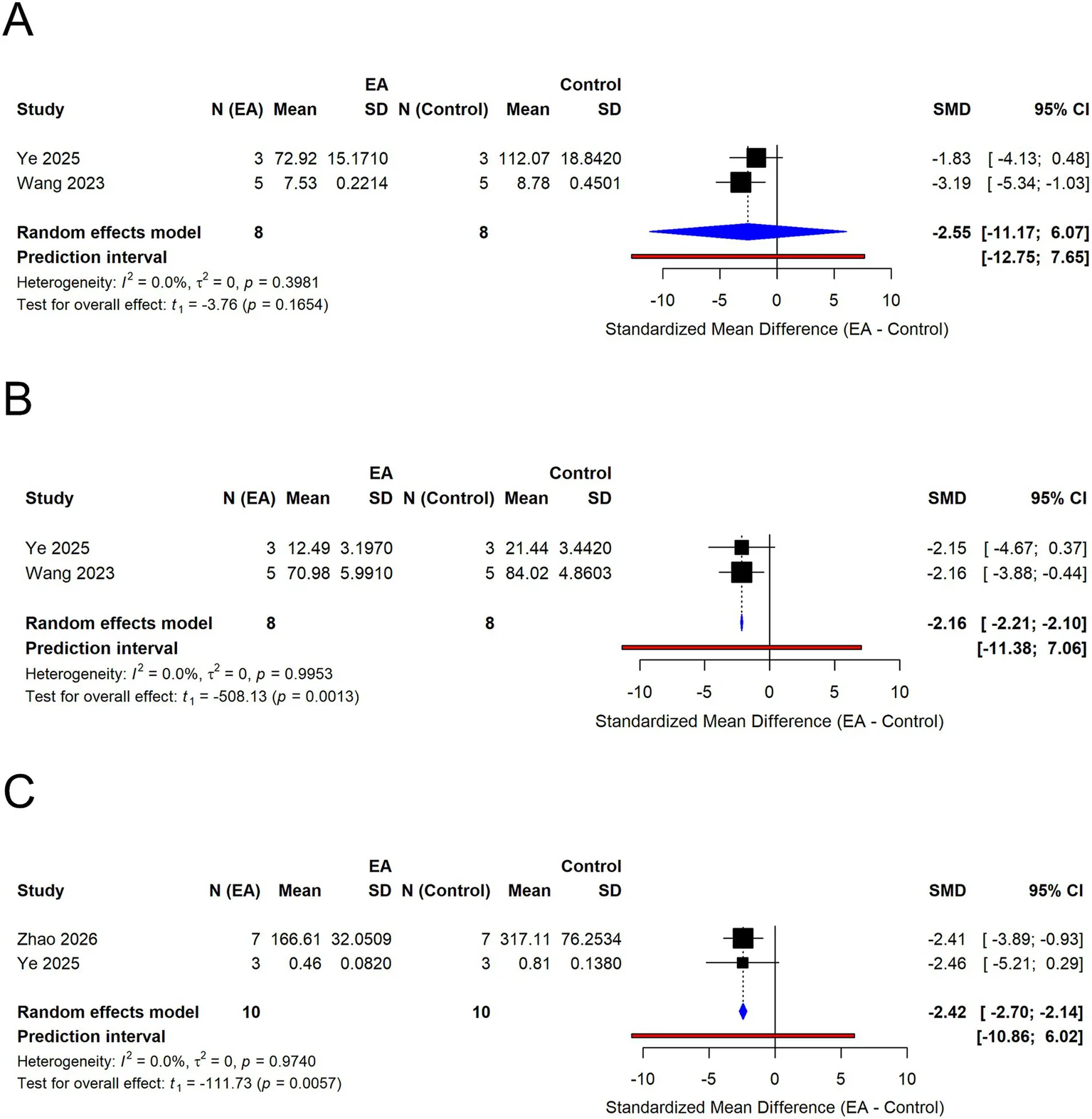
Meta-analysis of the effects of electroacupuncture on circulating gut barrier-related markers. **(A)** Forest plot of the pooled effect of electroacupuncture on diamine oxidase (DAO). **(B)** Forest plot of the pooled effect of electroacupuncture on D-lactate. **(C)** Forest plot of the pooled effect of electroacupuncture on lipopolysaccharide (LPS).

For circulating indicators of barrier injury, the pooled estimate for DAO did not reach statistical significance (2 studies, SMD = −2.550, 95% CI: −11.170 to 6.070, *p* = 0.165; *I*^2^ = 0.0%) (Figure 6A), whereas those for D-lactate (2 studies, SMD = −2.160, 95% CI: −2.210 to −2.100, *p* = 0.001; *I*^2^ = 0.0%) (Figure 6B) and LPS (2 studies, SMD = −2.420, 95% CI: −2.700 to −2.140, *p* = 0.006; *I*^2^ = 0.0%) (Figure 6C) reached statistical significance.

In addition to the markers described above, most other gut barrier-related outcomes were derived from single studies and were not included in the quantitative synthesis. Zhao Q. K. et al. (2026) reported that EA increased Muc2 expression, goblet cell density, UEA-1/α1,2-fucosylation, and Fut2 levels, and alleviated pathological injury in the small intestine; Ye et al. (2025) showed that EA pretreatment reduced ileal mucosal oedema, inflammatory cell infiltration, epithelial shedding, and subsequent fibrosis; Wang Y. L. et al. (2023) suggested that EA increased Claudin-1 levels and improved villus height and smooth muscle thickness; Chen Y. et al. (2025) showed that EA increased the villus-to-crypt (V/C) ratio and ameliorated morphological abnormalities in the small intestine, including villus shortening, crypt deepening, and epithelial shedding; Zhang et al. (2023) reported that EA reduced Chiu’s score and alleviated colonic mucosal injury; and histopathological findings based on haematoxylin and eosin staining in Wu Y. et al. (2026) showed that EA reduced colonic epithelial damage, glandular disorganization, and inflammatory cell infiltration. Zhao Q. K. et al. (2026) also reported that the effects of EA on gut barrier-related markers were attenuated or reversed following vagotomy.

#### 3.5.2 Gut inflammatory and immune-related markers

**Figure 7:**
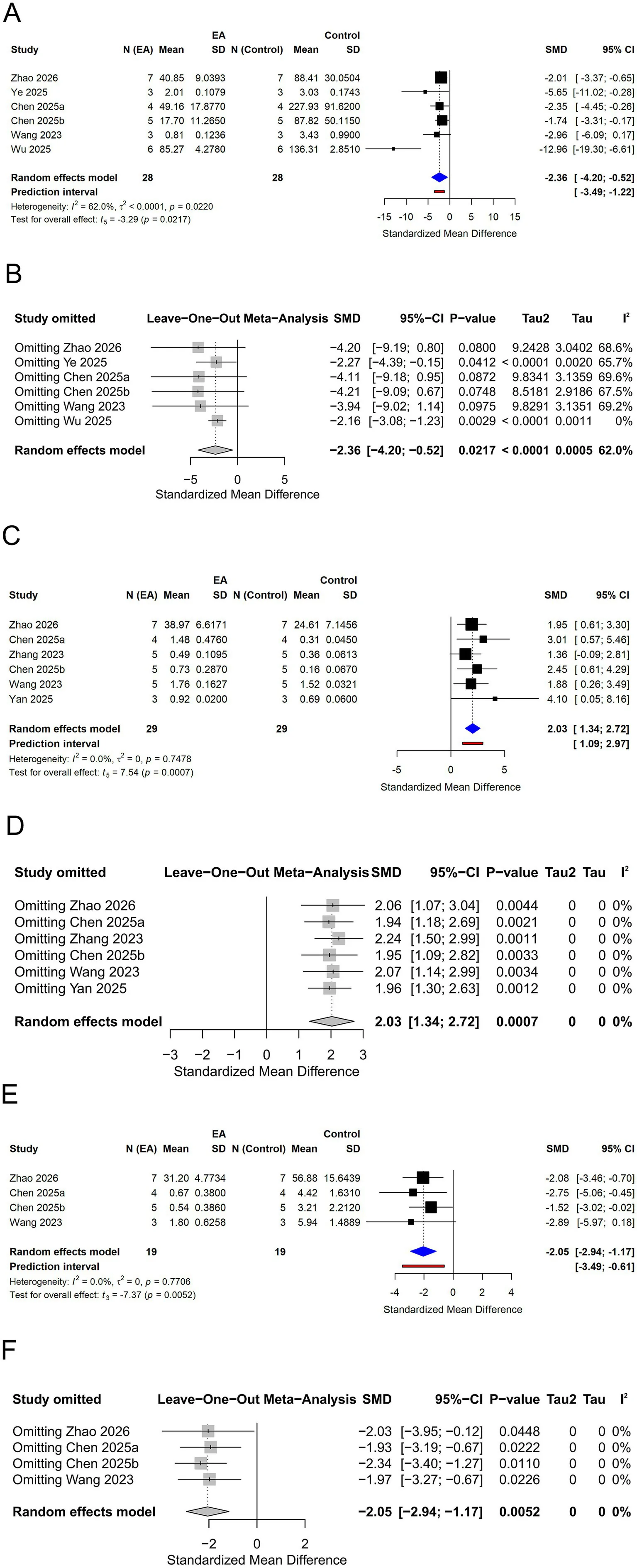
Meta-analysis of the effects of electroacupuncture on intestinal inflammatory cytokines. **(A)** Forest plot of the pooled effect of electroacupuncture on IL-1β. **(B)** Leave-one-out sensitivity analysis for IL-1β. **(C)** Forest plot of the pooled effect of electroacupuncture on IL-10. **(D)** Leave-one-out sensitivity analysis for IL-10. **(E)** Forest plot of the pooled effect of electroacupuncture on TNF-α. **(F)** Leave-one-out sensitivity analysis for TNF-α.

Three intestinal inflammatory cytokine markers were eligible for quantitative synthesis: IL-1β, IL-10, and TNF-α. Meta-analysis showed that EA significantly reduced intestinal IL-1β levels compared with the non-EA control group (6 studies, SMD = −2.360, 95% CI: −4.200 to −0.520, *p* = 0.022; *I*^2^ = 62.0%) (Figure 7A). Sensitivity analyses showed that the pooled IL-1β effect was not fully robust, as statistical significance disappeared after sequential exclusion of some studies (Figure 7B).

The pooled estimate for IL-10 showed that EA significantly increased intestinal IL-10 levels (6 studies, SMD = 2.030, 95% CI: 1.340 to 2.720, *p* < 0.001; *I*^2^ = 0.0%) (Figure 7C). Similarly, the pooled estimate for TNF-α showed that EA significantly reduced intestinal TNF-α levels (4 studies, SMD = −2.050, 95% CI: −2.940 to −1.170, *p* = 0.005; *I*^2^ = 0.0%) (Figure 7E). After sequential exclusion of individual studies, the pooled effects for both markers remained significant (Figures 7D,F).

**Figure 8:**
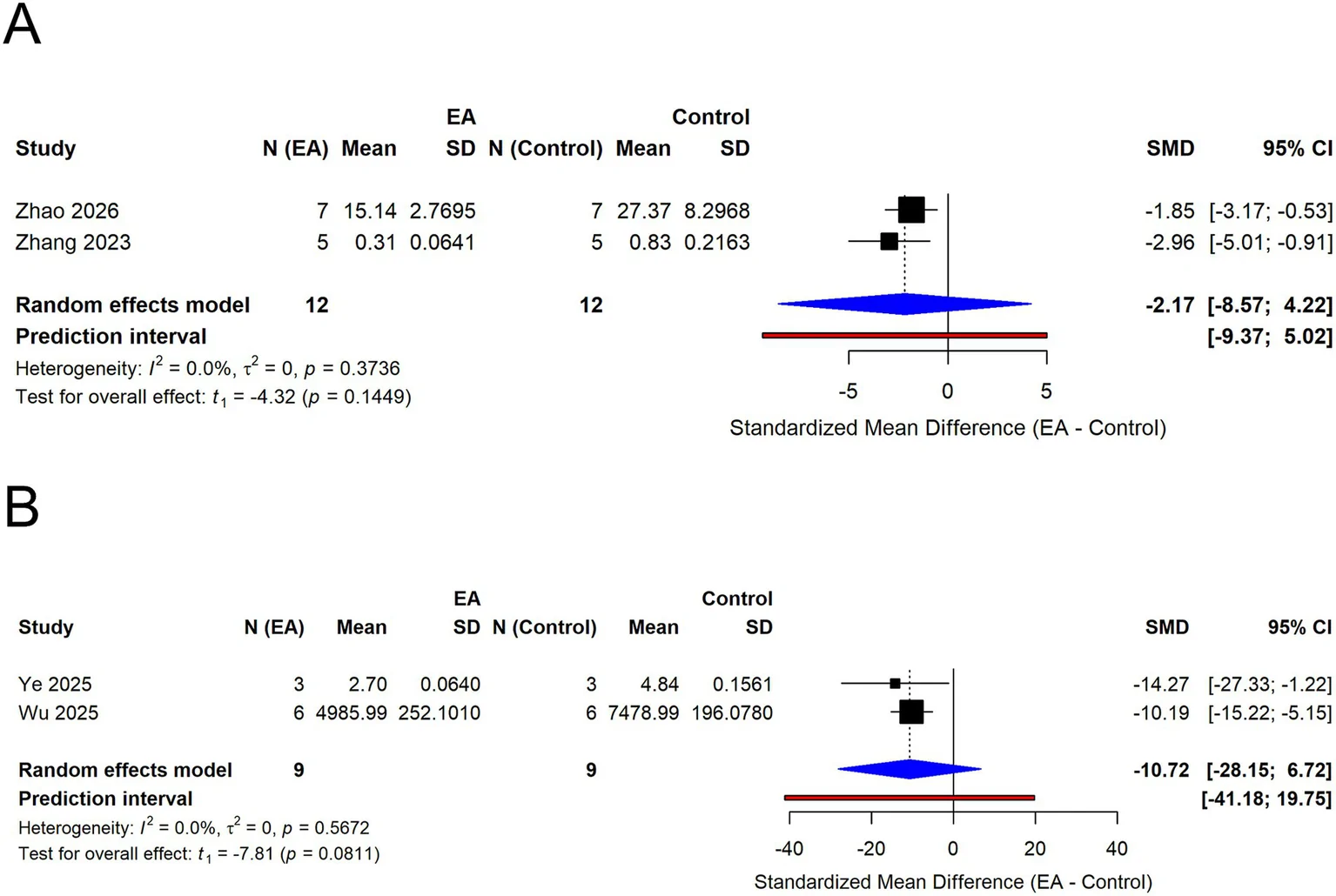
Meta-analysis of exploratory intestinal inflammatory markers. **(A)** Forest plot of the pooled effect of electroacupuncture on IL-17. **(B)** Forest plot of the pooled effect of electroacupuncture on NLRP3.

For exploratory inflammatory markers, the pooled estimates for IL-17 and NLRP3 did not reach statistical significance. The pooled effect estimate was SMD = −2.170 (95% CI: −8.570 to 4.220, *p* = 0.145) for IL-17 (Figure 8A) and SMD = −10.720 (95% CI: −28.150 to 6.720, *p* = 0.081) for NLRP3 (Figure 8B).

In addition to the cytokines described above, several studies also reported inflammation-related molecules that were not included in the quantitative synthesis. Ye et al. (2025) reported that EA pretreatment downregulated ileal TLR4, NF-κB p65, Caspase-1 p20, NLRP3, TXNIP, and IFN-γ, while upregulating TGF-β1, IL-4, and TRX1; Wang Y. L. et al. (2023) reported that EA reduced CXCL1 and CXCL2 expression in the small intestine. Histopathological findings based on haematoxylin and eosin staining in Ye et al. (2025), Chen Y. et al. (2025), and Wang Y. L. et al. (2023) showed that EA alleviated intestinal mucosal oedema, inflammatory cell infiltration, epithelial shedding, and villus injury.

**Figure 9:**
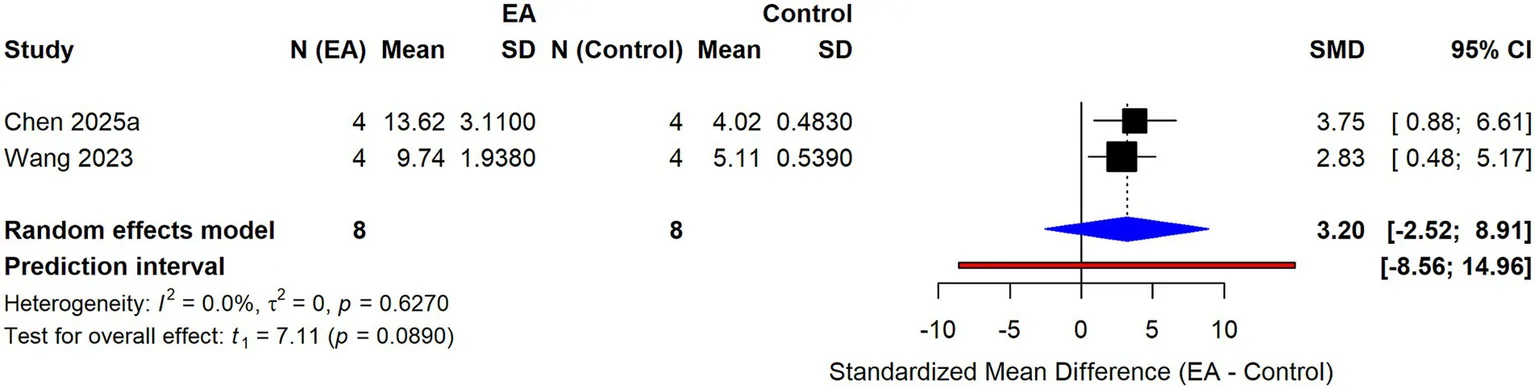
Meta-analysis of the exploratory effect of electroacupuncture on intestinal regulatory T cells (Treg).

Regarding immune-related markers, the exploratory pooled estimate for intestinal Treg cells (Foxp3+ cells as a percentage of CD45 + CD4+ cells) did not reach statistical significance (2 studies, SMD = 3.200, 95% CI: −2.520 to 8.910, *p* = 0.089) (Figure 9). Chen et al. (2025) reported that EA increased intestinal Foxp3 protein expression and acetylated Foxp3 levels, while inhibiting HDAC activity; Wang Y. L. et al. (2023) reported that EA increased the Treg/γδ T ratio while reducing CD4+ cells and TCRγδ/γδ T cell-related indicators.

#### 3.5.3 Gut microbiota-related findings

**Figure 10:**
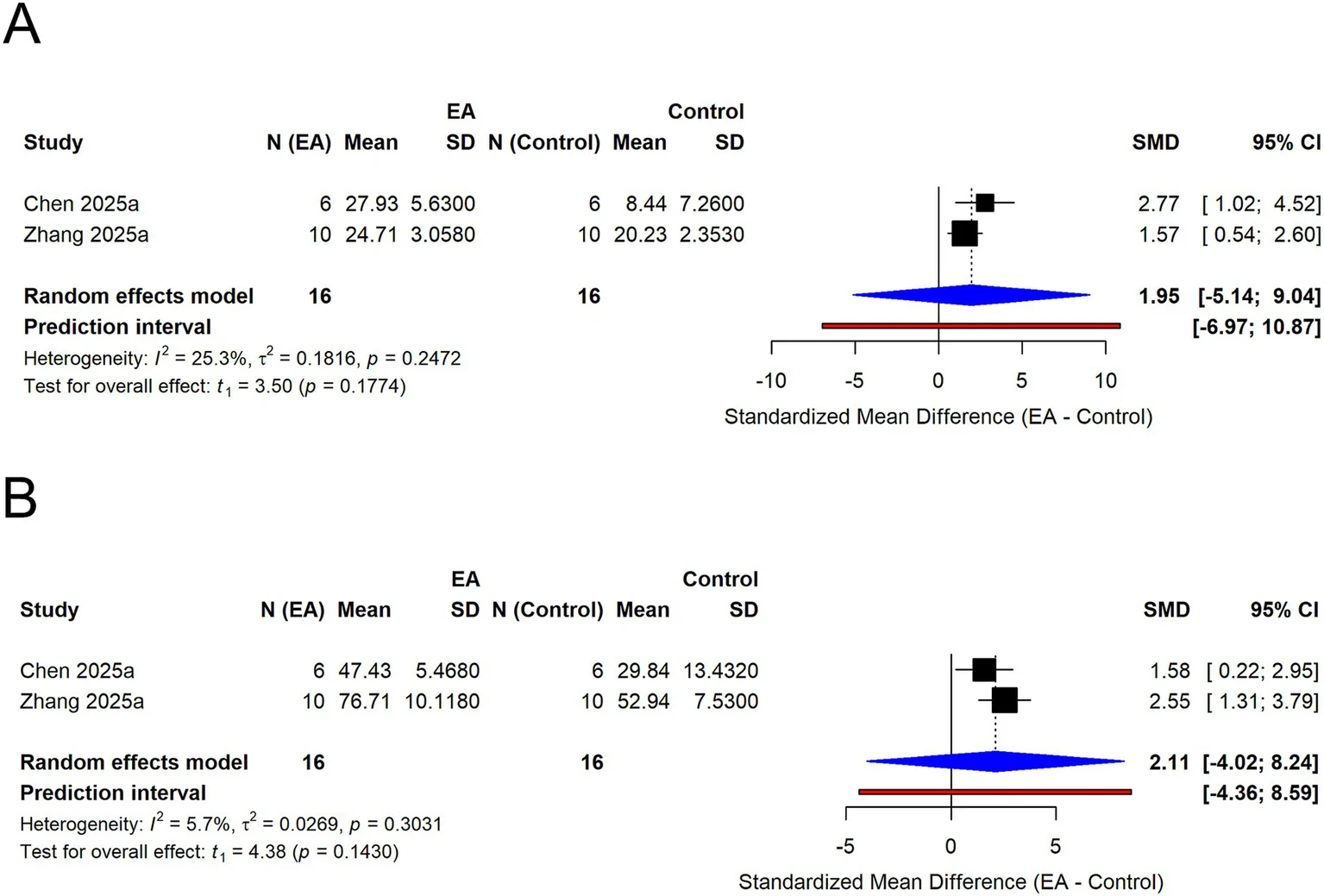
Meta-analysis of exploratory gut microbiota-related outcomes. **(A)** Forest plot of the pooled effect of electroacupuncture on Bacteroidota/Bacteroidetes abundance. **(B)** Forest plot of the pooled effect of electroacupuncture on Firmicutes abundance.

Among the available studies, gut microbiota-related outcomes were reported in highly heterogeneous formats, most commonly as α-diversity, β-diversity, differential taxonomic abundance, LEfSe, and functional pathway analyses. Accordingly, this section is primarily based on qualitative synthesis, with exploratory quantitative pooling performed only for Bacteroidota/Bacteroidetes and Firmicutes. Exploratory pooling showed no statistically significant effect of EA on either Bacteroidota/Bacteroidetes (2 studies: Chen Y. et al. (2025) and Zhang Y. et al. (2025); SMD = 1.950, 95% CI: −5.140 to 9.040, *p* = 0.177; *I*^2^ = 25.3%) (Figure 10A) or Firmicutes (2 studies: Chen Y. et al. (2025) and Zhang A. et al. (2025); SMD = 2.110, 95% CI: −4.020 to 8.240, *p* = 0.143; *I*^2^ = 5.7%) (Figure 10B).

Qualitative synthesis showed that studies such as Zhao Q. K. et al. (2026), Ye et al. (2025), Li S. et al. (2022), Zhang et al. (2023), and Chen Y. L. et al. (2025) reported reduced gut microbial diversity after stroke, whereas α-diversity indices, including ACE, Chao1, Sobs, Observed species, Shannon, and Simpson, were partially restored after EA. In β-diversity analyses, regardless of whether PCoA, PCA, NMDS, or UniFrac was used, the model group was clearly separated from the sham group, whereas the EA group clustered more closely with the sham group.

At the level of differential taxa, studies including Zhao Q. K. et al. (2026), Ye et al. (2025), Li S. et al. (2022), Zhang et al. (2023), Chen Y. L. et al. (2025), and Yan et al. (2025b) reported that EA increased the abundance of *Akkermansia*, *Lactobacillus*, Muribaculaceae, Lachnospiraceae, Prevotellaceae, *Butyrivibrio*, and *Bifidobacterium*, while decreasing the abundance of *Enterococcus*, *Escherichia*-*Shigella*, *Helicobacter*, *Fusobacterium*, and some proteobacteria-related taxa.

LEfSe and functional prediction analyses further supported these trends. Zhao Q. K. et al. (2026) reported enrichment of Prevotellaceae, *Butyrivibrio*, Lactobacillales, and Bacteroidales in the EA group, whereas Actinobacteria and Enterococcus were enriched in the MCAO group. Li S. et al. (2022) reported EA-associated shifts in the relative abundance of several endogenous bacterial taxa. Chen Y. L. et al. (2025) showed that the sham and EA groups were more enriched in Lachnospiraceae and Prevotellaceae, whereas the MCAO group was enriched in Enterobacteriaceae and Erysipelotrichaceae. Yan et al. (2025b) combined untargeted metabolomics with KEGG enrichment analysis and reported changes in differential metabolites and related pathways.

In addition, both Li S. et al. (2022) and Chen Y. L. et al. (2025) showed through faecal microbiota transplantation (FMT) that recipient animals receiving microbiota from EA-treated donors had better neurological function, smaller infarcts, and improved inflammatory status than those receiving microbiota from model donors. Chen Y. et al. (2025) also reported that microbiota changes related to short-chain fatty acid (SCFA) production occurred in parallel with increased Treg levels.

### 3.6 Certainty of evidence

The certainty of evidence varied across the quantitatively synthesized outcomes. Moderate-certainty evidence was identified for ZO-1, occludin, D-lactate, LPS, intestinal IL-10, and intestinal TNF-α. Low-certainty evidence was identified for infarct volume, DAO, intestinal IL-1β, IL-17, NLRP3, and intestinal Treg cells. The certainty of evidence for neurological function and the two exploratory gut microbiota-related outcomes was very low. Detailed outcome-level judgments and reasons for downgrading are presented in Table 2.

**Table 2:** GRADE certainty assessment of electroacupuncture-associated outcomes in experimental ischemic stroke.

Outcome	Studies	Animals (EA/control)	Effect estimate	Risk of bias	Inconsistency	Indirectness	Imprecision	Publication bias	Final certainty
Neurological function	11	101/101	SMD −2.49 (95% CI −3.50 to −1.47)	Serious	Serious	Not serious	Not serious	Serious	Very low
Infarct volume	9	54/54	SMD −3.08 (95% CI −4.22 to −1.95)	Serious	Serious	Not serious	Not serious	Not assessed	Low
ZO-1	4	16/16	SMD 3.07 (95% CI 0.74–5.39)	Not serious	Not serious	Not serious	Serious	Not assessed	Moderate
Occludin	4	16/16	SMD 3.72 (95% CI 2.17–5.26)	Not serious	Not serious	Not serious	Serious	Not assessed	Moderate
DAO	2	8/8	SMD −2.55 (95% CI −11.17–6.07)	Not serious	Not serious	Not serious	Very serious	Not assessed	Low
D-lactate	2	8/8	SMD −2.16 (95% CI −2.21−−2.10)	Not serious	Not serious	Not serious	Serious	Not assessed	Moderate
LPS	2	10/10	SMD −2.42 (95% CI −2.70−−2.14)	Not serious	Not serious	Not serious	Serious	Not assessed	Moderate
IL-1β	6	28/28	SMD −2.36 (95% CI −4.20−−0.52)	Not serious	Serious	Not serious	Serious	Not assessed	Low
IL-10	6	29/29	SMD 2.03 (95% CI 1.34–2.72)	Not serious	Not serious	Not serious	Serious	Not assessed	Moderate
TNF-α	4	19/19	SMD −2.05 (95% CI −2.94−−1.17)	Not serious	Not serious	Not serious	Serious	Not assessed	Moderate
IL-17	2	12/12	SMD −2.17 (95% CI −8.57–4.22)	Not serious	Not assessed	Not serious	Very serious	Not assessed	Low
NLRP3	2	9/9	SMD −10.72 (95% CI −28.15–6.72)	Not serious	Not assessed	Not serious	Very serious	Not assessed	Low
Treg cells	2	8/8	SMD 3.20 (95% CI −2.52–8.91)	Not serious	Not assessed	Not serious	Very serious	Not assessed	Low
Bacteroidota/Bacteroidetes	2	16/16	SMD 1.95 (95% CI −5.14–9.04)	Not serious	Not serious	Serious	Very serious	Not assessed	Very low
Firmicutes	2	16/16	SMD 2.11 (95% CI −4.02–8.24)	Not serious	Not serious	Serious	Very serious	Not assessed	Very low

## 4 Discussion

This study systematically integrated preclinical evidence on the effects of EA on neurological function, infarct-related outcomes, and brain–gut axis-related outcomes in animal models of ischemic stroke. The pooled results showed that EA was associated with improved neurological function and reduced infarct volume. In addition, EA was also associated with increased tight junction-related markers, reduced levels of certain pro-inflammatory factors, elevated anti-inflammatory factors, and partial restoration of gut microbial homeostasis. Overall, the observed neurological benefits of EA occurred alongside changes in several brain–gut axis-related domains, supporting a potential association between EA-related neuroprotection and modulation of brain–gut axis-related processes.

The GRADE assessment showed that the certainty of evidence ranged from moderate to very low across outcomes. Moderate-certainty evidence was identified for several intestinal barrier and inflammatory markers, whereas certainty was lower for infarct volume, exploratory inflammatory and immune outcomes, neurological function, and microbiota-related outcomes. The certainty was mainly limited by methodological concerns, inconsistency, imprecision, and possible publication bias. Accordingly, statistically significant pooled effects should be interpreted together with the corresponding certainty of evidence.

These findings are broadly consistent with previous systematic reviews and meta-analyses of acupuncture or EA in experimental ischemic stroke. Previous studies have suggested that acupuncture-related interventions can improve neurological function and reduce brain injury in animal models of stroke, and may also influence recovery-related processes such as neurogenesis and neuroplasticity (Lu et al., 2016; Wang et al., 2014). Compared with earlier reviews, the present study extends the scope of analysis to brain–gut axis-related outcomes, thereby providing a more comprehensive preclinical framework for understanding the multi-level effects of EA in ischemic stroke.

Mechanistically, peripheral sensory afferent activation may provide the initial neural input through which EA engages broader regulatory networks. Signals generated at the stimulated sites may be integrated through spinal and supraspinal pathways, including brainstem and hypothalamic autonomic centers. Studies outside stroke models have shown that stimulation site and intensity can determine the recruitment of distinct vagal–adrenal and spinal–sympathetic pathways (Li Y. W. et al., 2022; Liu et al., 2021; Liu et al., 2020). In experimental ischemic stroke, parasympathetic dysfunction attenuated EA-associated improvements in neurological function, cerebral perfusion, infarct volume, and inflammatory responses, supporting a potential role for autonomic regulation in its neuroprotective effects (Chi et al., 2018). These findings support a broader working model in which sensory afferent and central/autonomic regulation may precede or occur alongside neuroimmune modulation, whereas peripheral immune and intestinal changes may arise as downstream adaptations, parallel responses, or reciprocal feedback signals. However, this complete sequence was not directly examined in most of the included studies and should not be interpreted as an established linear pathway.

Beyond autonomic and brain–gut pathways, direct central neuroprotective and repair mechanisms may also contribute to the effects of EA (Lin et al., 2026; Zhao M. et al., 2026). Experimental evidence suggests that EA pretreatment can modulate microglial activation-related responses and inhibit the TLR4/NF-κB/TXNIP/NLRP3 athway, thereby attenuating post-ischemic neuroinflammation (Ye et al., 2024). EA has also been reported to reduce oxidative stress through Nrf2-related antioxidant signaling (Pu et al., 2025). More recent evidence indicates that EA may promote post-ischemic angiogenesis through an FTO-dependent, m6A-mediated miR-214-3p/EZH2/eNOS pathway (Song et al., 2026). During the recovery phase, EA may support neurogenesis and BDNF/TrkB-related synaptic plasticity (Yu et al., 2024). These central mechanisms may operate alongside autonomic, immune, and intestinal responses and jointly contribute to neurological recovery.

Importantly, the primary pathological event remains cerebral arterial occlusion and reduced cerebral perfusion, followed by energy failure and neuronal injury (Campbell et al., 2019). Brain–gut axis-related changes are more plausibly positioned within the secondary pathophysiology of stroke, in which central autonomic and neuroimmune signals may contribute to intestinal barrier disruption, peripheral immune dysregulation, and microbial and metabolic alterations. These peripheral changes may subsequently feedback to influence neuroinflammation, systemic complications, and functional recovery (Tiedt et al., 2022; Yang et al., 2022). Accordingly, brain–gut axis-related processes should be interpreted as potential modifiers of disease progression and recovery rather than as primary causes of stroke.

Within this secondary-injury and recovery framework, brain–gut interactions may represent one component of the systemic responses associated with EA. Benakis et al. (2016) showed that the gut microbiota can influence stroke outcomes by modulating intestinal γδ T cells, whereas Crapser et al. reported increased intestinal permeability and bacterial translocation after stroke (Crapser et al., 2016). Previous reviews have further suggested that post-stroke microbial dysbiosis, gut barrier disruption, and immune activation collectively contribute to brain–gut axis dysfunction (Hu et al., 2022; Peh et al., 2022). Taken together, these findings support the biological relevance of brain–gut interactions after stroke, while indicating that intestinal changes should be interpreted within a broader neural, autonomic, and immune regulatory network rather than as the initial direct target of EA.

A related extension of this systemic framework is the oral–systemic–brain axis, which may represent an additional, but currently untested, pathway relevant to EA-related neuroprotection. Periodontitis has been associated with an increased risk of ischemic stroke, and salivary microbiome profiles have been linked to stroke severity and 90-day prognosis (Leira et al., 2017; Sun et al., 2023). Experimental evidence further showed that *Porphyromonas gingivalis*-associated periodontitis aggravated infarct volume, sensorimotor deficits, blood–brain barrier disruption, systemic inflammation, and brain neutrophil infiltration after cerebral ischemia (Diallo et al., 2026). Notably, EA at ST36 has been reported to reduce periodontal inflammatory-cell infiltration and pro-inflammatory cytokine expression while altering the oral microbiota in a mouse model of ligature-induced periodontitis (Liu et al., 2023). These findings provide indirect biological plausibility that EA might influence oral immune–microbial processes with downstream systemic and cerebral consequences. However, none of the ischemic stroke studies included in the present review evaluated periodontal status, oral microbiota, oral pathogen burden, or related circulating inflammatory signals. Therefore, the oral–systemic–brain axis should be considered a complementary hypothesis rather than an established mechanism of EA-related neuroprotection.

Within the brain–gut component of this broader systemic framework, improvement in intestinal barrier integrity may represent one of the processes associated with the broader effects of EA after stroke. However, because most studies assessed intestinal and neurological outcomes concurrently, the temporal sequence and mediating role of these barrier-related changes remain uncertain. Therefore, intestinal barrier restoration should be interpreted as a potential contributing process rather than an established upstream mechanism of EA-related neuroprotection (Yong et al., 2023). Previous studies have shown that disruption of the intestinal barrier after ischemic stroke can promote bacterial translocation, endotoxemia, and an increased risk of infection, thereby further aggravating neuroinflammation and poor prognosis (Clottes et al., 2023; Kurita et al., 2020; Wei et al., 2021). In the present study, EA was associated with increased levels of ZO-1 and Ocln and decreased levels of D-lactate and LPS. These findings are broadly consistent with the emerging view that intestinal barrier integrity may serve as a therapeutic target after stroke (Cuartero et al., 2024; Huang et al., 2022; Wang J. et al., 2023). From a biological perspective, preservation of epithelial integrity could reduce the translocation of bacterial components and microbial metabolites from the intestinal lumen into the circulation, thereby limiting the amplification of peripheral inflammatory responses after stroke (Long et al., 2022; Sharma et al., 2021; Yamashiro et al., 2021). However, because the included studies generally assessed intestinal and neurological outcomes at similar time points and did not formally test mediation, the current evidence does not demonstrate that intestinal barrier restoration precedes or mediates EA-related neurological improvement. Intestinal barrier improvement should therefore be interpreted as one of several concurrent or potentially contributing systemic changes associated with EA after ischemic stroke.

With respect to inflammatory and immune regulation, the present study found that EA was associated with reduced levels of pro-inflammatory factors such as IL-1β and TNF-α, as well as increased IL-10, whereas the available evidence remains relatively limited for IL-17, NLRP3, and Treg/Foxp3-related markers. Overall, these findings are consistent with current evidence that post-stroke intestinal abnormalities may further influence peripheral immune activation, amplification of intestinal inflammation, and secondary brain injury (Chidambaram et al., 2022; Yang et al., 2022). In particular, recent studies on the brain–gut axis have increasingly emphasized the bridging role of intestinal immune cells in stroke, including the potential influence of Treg cells, γδ T cells, and other peripheral immune cell populations on the progression of brain injury and functional recovery (Tiedt et al., 2022; Zhou et al., 2023). The effects of EA on Foxp3, Treg cells, and related immune homeostasis suggest that its role may extend beyond merely suppressing inflammation and may be more appropriately understood as modulating immune balance. Recent studies have reported that EA-related increases in Treg responses occur alongside changes in gut microbiota and improved neurological outcomes, suggesting potential interactions among these processes (Chen Y. L. et al., 2025; Zhang A. et al., 2025; Zhang et al., 2023). Therefore, the effects of EA on inflammatory and immune-related markers may not be limited to anti-inflammatory actions alone, but may also involve modulation of immune homeostasis.

Gut microbiota-related findings should be interpreted cautiously. Taxonomic profiles in animal studies may be influenced by housing environment, cage conditions, diet, host characteristics, disease stage, sampling time, and analytical methods. Recent experimental studies have shown that the immediate laboratory environment, control diet, and temporal host factors can substantially alter murine gut microbial composition (Dowden et al., 2024; Ericsson et al., 2025; Guo et al., 2024). Therefore, an increase or decrease in an individual taxon should not automatically be interpreted as evidence of a beneficial or detrimental function. In the present review, the more informative findings were the links between microbial alterations and host-level responses, including intestinal barrier integrity, inflammatory and immune regulation, SCFA- and tryptophan-related metabolism, and the effects observed after FMT. Recent transplantation and multi-omic evidence also suggests that the influence of the gut microbiota on stroke outcomes may depend on microbial metabolites and host inflammatory or barrier responses rather than taxonomic composition alone (Wu G. et al., 2026). Accordingly, the observed taxonomic shifts should be regarded as exploratory ecological signatures that require functional validation rather than direct evidence of microbiota-mediated recovery.

**Figure 11:**
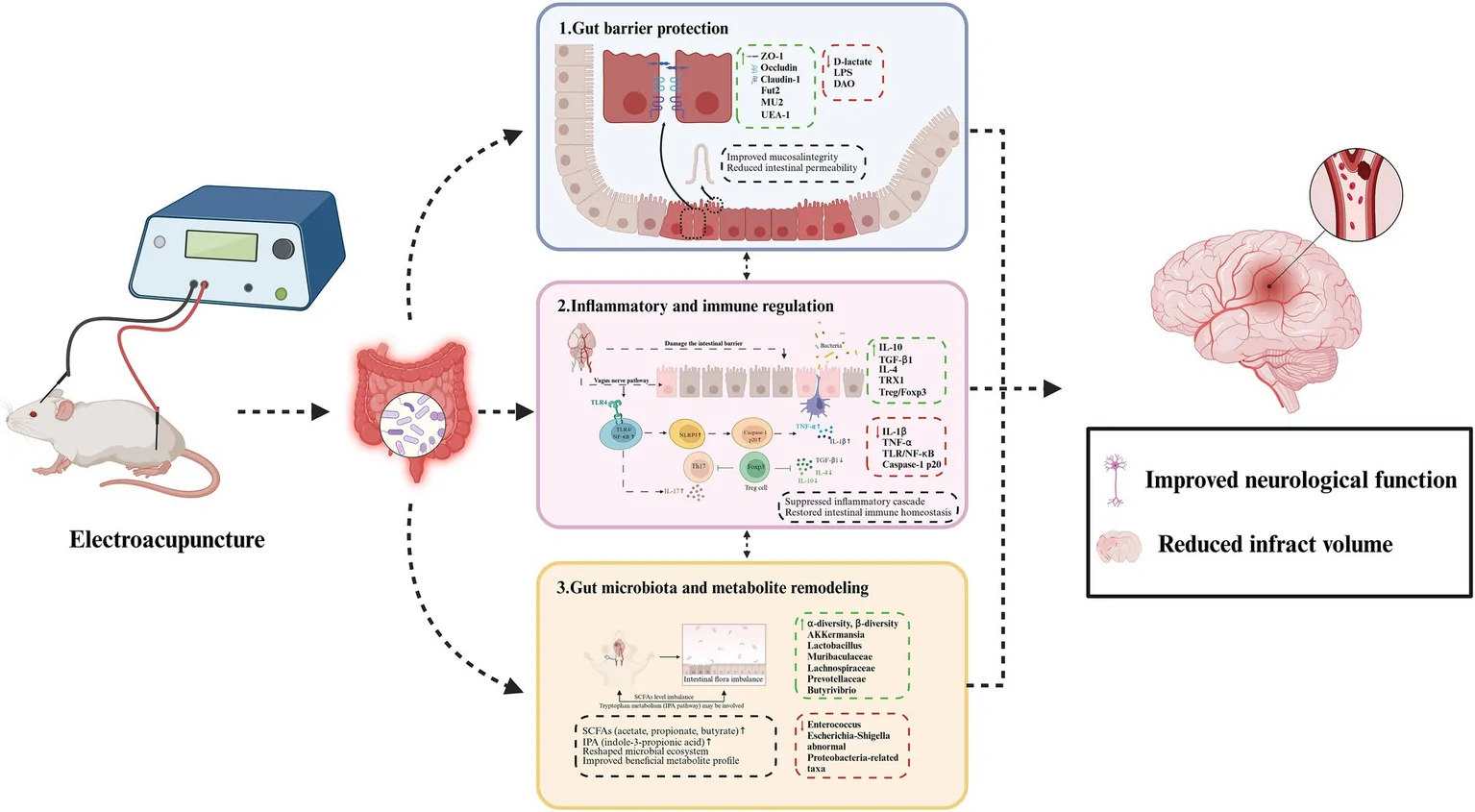
Conceptual framework of electroacupuncture-associated neurological and brain–gut axis-related changes in experimental ischemic stroke.

Taken together, the available evidence suggests that EA-related neurological improvement is accompanied by changes in intestinal barrier integrity, inflammatory and immune responses, and microbial and metabolic profiles. These findings may be summarized within a descriptive framework linking intestinal barrier changes, immune-inflammatory regulation, and microbiota–metabolite interactions associated with EA after ischemic stroke (Figure 11).

The pooled findings should be interpreted in light of the heterogeneity across experimental conditions. Variations in animal background, MCAO procedures, ischemia duration, filament characteristics, and anaesthesia may influence infarct severity, blood–brain barrier injury, and functional outcomes (Mendes et al., 2026). Differences in intervention timing, acupoint selection, waveform, stimulation parameters, and treatment duration may also affect the magnitude of EA-related effects (Guo Y. et al., 2025; Yang et al., 2026). In addition, variations in outcome measures and assessment time points may contribute to measurement heterogeneity. Therefore, although SMDs permit statistical pooling across different scales, the pooled estimates should be interpreted as average effects across heterogeneous experimental protocols rather than evidence for a single standardized or optimal EA regimen.

Several limitations of this study should be acknowledged. First, although the primary outcome analyses supported protective effects of EA on neurological function and infarct-related outcomes, some heterogeneity remained across several outcomes. This heterogeneity may be related to differences in animal species and strains, ischemia models and durations, intervention timing, acupoint combinations, EA parameters, and outcome measurement methods. Previous preclinical reviews have likewise identified such methodological differences as common sources of heterogeneity in meta-analyses of animal studies (Yang et al., 2026). Second, the funnel plot and Egger’s test for neurological outcomes suggested possible small-study effects or publication bias. In addition, methodological reporting of the included studies was generally insufficient with respect to randomization, allocation concealment, blinding, and selective reporting, which may have affected the precision of effect estimation. Third, some data were extracted from figures, and conversion using WebPlotDigitizer 4.3 may have introduced measurement error, particularly when the original image quality was suboptimal. Fourth, many brain–gut axis-related indicators were derived from only a small number of studies, and some exploratory outcomes could be pooled from only two studies, limiting the stability of the corresponding conclusions. Fifth, the initial biological targets of EA, as well as the temporal direction and mediating role of brain–gut axis-related changes, remain uncertain. Most included studies assessed neurological outcomes and gut-related parameters at similar time points but did not simultaneously examine peripheral sensory afferent activation, central or autonomic neural regulation, neuroimmune responses, and intestinal changes. Only a small number employed mechanistic interventions such as FMT or vagotomy. Consequently, it remains unclear whether gut-related changes precede neurological improvement, occur secondary to central neural or neuroimmune effects, or develop concurrently with these responses. The available evidence is therefore insufficient to determine whether brain–gut axis modulation is a primary mediator of EA-related neuroprotection. Sixth, most included studies used male animals, and substantial variation existed in EA stimulation parameters, acupoint combinations, and treatment duration. In addition, needle specifications, insertion depth, electrode configuration, pulse width, restraint or anaesthesia during stimulation, and adverse-event monitoring were not consistently reported in the original studies, limiting full procedural reproducibility. These factors limit the generalizability of the findings and the comparability of parameter-specific effects. Finally, all included studies were conducted in animal models, predominantly using young, healthy male rodents. These models do not adequately reflect the advanced age, sex diversity, vascular risk factors, comorbidities, stroke heterogeneity, and concurrent treatments commonly encountered in patients. Moreover, many studies used EA pretreatment or initiated treatment shortly after modelling, which may not correspond to clinically feasible treatment windows. Therefore, the present findings support biological activity and mechanistic plausibility but should not be interpreted as evidence of clinical efficacy.

Despite these limitations, the present study provides a more integrative perspective on the effects associated with EA in ischemic stroke. Although ischemic stroke is initiated by a focal cerebral vascular event, subsequent pathological changes may extend beyond the brain and involve bidirectional interactions between the brain and the gut. Within this secondary systemic framework, EA-related benefits may be associated with modulation of multiple interrelated processes, including neural and autonomic regulation, intestinal barrier integrity, inflammatory and immune responses, and gut microbial homeostasis. This perspective provides a conceptual link between traditional acupuncture interventions and the systemic pathophysiology of stroke, while its clinical relevance requires further validation. Clinical investigation of EA for post-stroke outcomes is emerging, although direct clinical evidence regarding its effects on brain–gut axis-related processes remains lacking.

Future preclinical studies should include aged animals, both sexes, and models with clinically relevant comorbidities, such as hypertension, diabetes, or metabolic dysfunction. EA should be evaluated within clinically feasible post-stroke treatment windows and, where appropriate, in combination with reperfusion therapy, pharmacological treatment, and rehabilitation. Greater attention should also be given to parameter–response relationships, treatment safety, and the durability of neurological and systemic effects. Serial assessments and targeted interventions, including vagal modulation, microbiota manipulation, and pathway-blocking experiments, may help clarify the temporal and causal relationships among central neural responses, intestinal barrier changes, immune regulation, microbial metabolites, and neurological recovery. These steps are necessary before the current preclinical findings can provide a reliable basis for clinical trial design.

In summary, EA was associated with improved neurological outcomes and concurrent changes in intestinal barrier integrity, inflammatory and immune responses, and gut microbiota-related profiles in animal models of ischemic stroke. These findings suggest that brain–gut axis-related processes may be associated with the multifaceted effects of EA after ischemic stroke. However, the current evidence does not establish brain–gut axis modulation as the primary mediator of EA-induced neuroprotection, and the temporal and causal relationships among these changes remain to be clarified through further mechanistic studies.

## Data Availability

The original contributions presented in the study are included in the article/Supplementary material, further inquiries can be directed to the corresponding author.
