# Supplementary material for: Effects of electroacupuncture on neurological outcomes and brain–gut axis-associated changes in experimental ischemic stroke models: a systematic review and meta-analysis
Source: Front Neurosci. 2026 Aug 25;20:1893796. doi: 10.3389/fnins.2026.1893796 (PMC13550116; doi:10.3389/fnins.2026.1893796)
Supplement: Supplementary file 2 [file Supplementary_file_2.docx]

**Supplementary File 2 Search Strategy**

**Pubmed**

| # | searches | resultes |
| --- | --- | --- |
| 1 | "Electroacupuncture"[Mesh]  OR electroacupuncture[Title/Abstract]  OR electro-acupuncture[Title/Abstract]  OR "electric acupuncture"[Title/Abstract] | 9,298 |
| 2 | "Stroke"[Mesh]  OR "Brain Ischemia"[Mesh]  OR stroke[Title/Abstract]  OR "ischemic stroke"[Title/Abstract]  OR "cerebral ischemia"[Title/Abstract]  OR "brain ischemia"[Title/Abstract]  OR "cerebral infarction"[Title/Abstract]  OR "cerebral infarct*"[Title/Abstract]  OR "ischemic brain injury"[Title/Abstract]  OR "middle cerebral artery occlusion"[Title/Abstract]  OR MCAO[Title/Abstract]  OR "MCAO/R"[Title/Abstract]  OR "I/R"[Title/Abstract]  OR CIRI[Title/Abstract] | 497,136 |
| 3 | "Gastrointestinal Microbiome"[Mesh]  OR "gut microbiota"[Title/Abstract]  OR "gut microbiome"[Title/Abstract]  OR "intestinal microbiota"[Title/Abstract]  OR "gut flora"[Title/Abstract]  OR "gut-brain axis"[Title/Abstract]  OR "brain-gut axis"[Title/Abstract]  OR dysbiosis[Title/Abstract]  OR "intestinal barrier"[Title/Abstract]  OR "gut barrier"[Title/Abstract]  OR "intestinal permeability"[Title/Abstract]  OR "tight junction"[Title/Abstract]  OR "ZO-1"[Title/Abstract]  OR occludin[Title/Abstract]  OR claudin*[Title/Abstract]  OR DAO[Title/Abstract]  OR "diamine oxidase"[Title/Abstract]  OR "D-lactate"[Title/Abstract]  OR LPS[Title/Abstract]  OR lipopolysaccharide[Title/Abstract]  OR "short-chain fatty acids"[Title/Abstract]  OR SCFAs[Title/Abstract]  OR TMAO[Title/Abstract]  OR "trimethylamine N-oxide"[Title/Abstract]  OR IPA[Title/Abstract]  OR "indole-3-propionic acid"[Title/Abstract]  OR "16S rRNA"[Title/Abstract]  OR LEfSe[Title/Abstract] | 417,161 |
| 4 | animals[MeSH Terms] | 28,842,216 |
| 5 | #1 AND (#2 OR #3) AND #4 | 29 |

**Embase**

| # | searches | resultes |
| --- | --- | --- |
| 1 | 'electroacupuncture'/exp  OR electroacupuncture:ti,ab,kw  OR 'electro-acupuncture':ti,ab,kw  OR 'electric acupuncture':ti,ab,kw | 14,081 |
| 2 | 'stroke'/exp  OR 'brain ischemia'/exp  OR stroke:ti,ab,kw  OR 'ischemic stroke':ti,ab,kw  OR 'cerebral ischemia':ti,ab,kw  OR 'brain ischemia':ti,ab,kw  OR 'cerebral infarction':ti,ab,kw  OR 'cerebral infarct*':ti,ab,kw  OR 'ischemic brain injury':ti,ab,kw  OR 'middle cerebral artery occlusion':ti,ab,kw  OR MCAO:ti,ab,kw  OR 'MCAO/R':ti,ab,kw  OR 'I/R':ti,ab,kw  OR CIRI:ti,ab,kw | 914,924 |
| 3 | 'gastrointestinal microbiome'/exp  OR 'gut microbiota':ti,ab,kw  OR 'gut microbiome':ti,ab,kw  OR 'intestinal microbiota':ti,ab,kw  OR 'gut flora':ti,ab,kw  OR 'gut-brain axis':ti,ab,kw  OR 'brain-gut axis':ti,ab,kw  OR dysbiosis:ti,ab,kw  OR 'intestinal barrier':ti,ab,kw  OR 'gut barrier':ti,ab,kw  OR 'intestinal permeability':ti,ab,kw  OR 'tight junction':ti,ab,kw  OR 'ZO-1':ti,ab,kw  OR occludin:ti,ab,kw  OR claudin*:ti,ab,kw  OR DAO:ti,ab,kw  OR 'diamine oxidase':ti,ab,kw  OR 'D-lactate':ti,ab,kw  OR LPS:ti,ab,kw  OR lipopolysaccharide:ti,ab,kw  OR 'short-chain fatty acids':ti,ab,kw  OR SCFAs:ti,ab,kw  OR TMAO:ti,ab,kw  OR 'trimethylamine N-oxide':ti,ab,kw  OR IPA:ti,ab,kw  OR 'indole-3-propionic acid':ti,ab,kw  OR '16S rRNA':ti,ab,kw  OR LEfSe:ti,ab,kw | 554,726 |
| 4 | 'animal experiment'/exp  OR animal*:ti,ab,kw  OR rat:ti,ab,kw  OR rats:ti,ab,kw  OR mouse:ti,ab,kw  OR mice:ti,ab,kw | 6,097,012 |
| 7 | #1 AND (#2 OR #3) AND #4 | 34 |

**Web of science**

| # | searches | resultes |
| --- | --- | --- |
| 1 | TS=  ("electroacupuncture" OR "electro-acupuncture" OR "electric acupuncture") | 8,216 |
| 2 | TS=  (  "stroke"  OR "brain ischemia"  OR "ischemic stroke"  OR "cerebral ischemia"  OR "brain ischemia"  OR "cerebral infarction"  OR "cerebral infarct*"  OR "ischemic brain injury"  OR "middle cerebral artery occlusion"  OR MCAO  OR "MCAO/R"  OR "I/R"  OR CIRI  ) | 523,205 |
| 3 | TS=(  "gut microbiota"  OR "gut microbiome"  OR "intestinal microbiota"  OR "gut flora"  OR "gut-brain axis"  OR "brain-gut axis"  OR dysbiosis  OR "intestinal barrier"  OR "gut barrier"  OR "intestinal permeability"  OR "tight junction"  OR "ZO-1"  OR occludin  OR claudin*  OR DAO  OR "diamine oxidase"  OR "D-lactate"  OR LPS  OR lipopolysaccharide  OR "short-chain fatty acids"  OR SCFAs  OR TMAO  OR "trimethylamine N-oxide"  OR IPA  OR "indole-3-propionic acid"  OR "16S rRNA"  OR LEfSe  ) | 462,111 |
| 4 | TS=(  animal*  OR rat  OR rats  OR mouse  OR mice  ) | 3,608,617 |
| 5 | #1 AND #2 AND #3 AND #4 | 31 |

**Cochrane library**

| # | searches | resultes |
| --- | --- | --- |
| 1 | MeSH descriptor: [Electroacupuncture] explode all trees | 1257 |
| 2 | electroacupuncture:ti,ab,kw | 4309 |
| 3 | electro-acupuncture:ti,ab,kw | 755 |
| 4 | "electric acupuncture":ti,ab,kw | 148 |
| 5 | #1 OR #2 OR #3 OR #4 | 4451 |
| 6 | MeSH descriptor: [Stroke] explode all trees | 19308 |
| 7 | MeSH descriptor: [Brain Ischemia] explode all trees | 5934 |
| 8 | stroke:ti,ab,kw | 81165 |
| 9 | "ischemic stroke":ti,ab,kw | 16165 |
| 10 | "cerebral ischemia":ti,ab,kw | 1443 |
| 11 | "brain ischemia":ti,ab,kw | 6607 |
| 12 | "cerebral infarction":ti,ab,kw | 5318 |
| 13 | "cerebral infarct":ti,ab,kw | 216 |
| 14 | "ischemic brain injury":ti,ab,kw | 134 |
| 15 | "middle cerebral artery occlusion":ti,ab,kw | 249 |
| 16 | MCAO:ti,ab,kw | 32 |
| 17 | "MCAO/R":ti,ab,kw | 1 |
| 18 | "I/R":ti,ab,kw | 440 |
| 19 | CIRI:ti,ab,kw | 8 |
| 20 | #6 OR #7 OR #8 OR #9 OR #10 OR #11 OR #12 OR #13 OR #14 OR #15 OR #16 OR #17 OR #18 OR #19 | 86023 |
| 21 | MeSH descriptor: [Gastrointestinal Microbiome] explode all trees | 2258 |
| 22 | "gut microbiota":ti,ab,kw | 5747 |
| 23 | "gut microbiome":ti,ab,kw | 2843 |
| 24 | "intestinal microbiota":ti,ab,kw | 1509 |
| 25 | "gut flora":ti,ab,kw | 304 |
| 26 | "gut-brain axis":ti,ab,kw | 437 |
| 27 | "brain-gut axis":ti,ab,kw | 252 |
| 28 | dysbiosis:ti,ab,kw | 1603 |
| 29 | "intestinal barrier":ti,ab,kw | 637 |
| 30 | "gut barrier":ti,ab,kw | 419 |
| 31 | "intestinal permeability":ti,ab,kw | 965 |
| 32 | "tight junction":ti,ab,kw | 225 |
| 33 | "ZO-1":ti,ab,kw | 71 |
| 34 | occludin:ti,ab,kw | 80 |
| 35 | claudin*:ti,ab,kw | 226 |
| 36 | DAO:ti,ab,kw | 322 |
| 37 | "diamine oxidase":ti,ab,kw | 239 |
| 38 | "D-lactate":ti,ab,kw | 119 |
| 39 | LPS:ti,ab,kw | 2585 |
| 40 | lipopolysaccharide:ti,ab,kw | 1936 |
| 41 | "short-chain fatty acids":ti,ab,kw | 1426 |
| 42 | SCFAs:ti,ab,kw | 574 |
| 43 | TMAO:ti,ab,kw | 305 |
| 44 | "trimethylamine N-oxide":ti,ab,kw | 283 |
| 45 | IPA:ti,ab,kw | 548 |
| 46 | "indole-3-propionic acid":ti,ab,kw | 15 |
| 47 | "16S rRNA":ti,ab,kw | 1 |
| 48 | LEfSe:ti,ab,kw | 94 |
| 49 | #21 OR #22 OR #23 OR #24 OR #25 OR #26 OR #27 OR #28 OR #29 OR #30 OR #31 OR #32 OR #33 OR #34 OR #35 OR #36 OR #37 OR #38 OR #39 OR #40 OR #41 OR #42 OR #43 OR #44 OR #45 OR #46 OR #47 OR #48 | 16664 |
| 50 | animal*:ti,ab,kw OR rat:ti,ab,kw OR rats:ti,ab,kw OR mouse:ti,ab,kw OR mice:ti,ab,kw | 45153 |
| 51 | #5 AND #20 AND #49 AND #50 | 0 |

**Scopus**

| # | searches | resultes |
| --- | --- | --- |
| 1 | TITLE-ABS-KEY(  electroacupuncture  OR "electro-acupuncture"  OR "electric acupuncture"  ) | 14,181 |
| 2 | TITLE-ABS-KEY(  stroke  OR "brain ischemia"  OR "ischemic stroke"  OR "cerebral ischemia"  OR "brain ischemia"  OR "cerebral infarction"  OR "cerebral infarct*"  OR "ischemic brain injury"  OR "middle cerebral artery occlusion"  OR MCAO  OR "MCAO/R"  OR "I/R"  OR CIRI  ) | 764,450 |
| 3 | TITLE-ABS-KEY(  "gut microbiota"  OR "gut microbiome"  OR "intestinal microbiota"  OR "gut flora"  OR "gut-brain axis"  OR "brain-gut axis"  OR dysbiosis  OR "intestinal barrier"  OR "gut barrier"  OR "intestinal permeability"  OR "tight junction"  OR "ZO-1"  OR occludin  OR claudin*  OR DAO  OR "diamine oxidase"  OR "D-lactate"  OR LPS  OR lipopolysaccharide  OR "short-chain fatty acids"  OR SCFAs  OR TMAO  OR "trimethylamine N-oxide"  OR IPA  OR "indole-3-propionic acid"  OR "16S rRNA"  OR LEfSe  ) | 576,340 |
| 4 | TITLE-ABS-KEY(  animal*  OR rat  OR rats  OR mouse  OR mice  ) | 9,625,332 |
| 5 | #1 AND #2 AND #3 AND #4 | 40 |

**CNKI**

| # | searches | resultes |
| --- | --- | --- |
| 1 | SU=('电针'+'电针疗法'+'电针预处理'+'电针治疗')  AND  SU=('缺血性脑卒中'+'缺血性卒中'+'脑缺血'+'脑梗死'+'脑梗塞'+'脑缺血再灌注'+'脑缺血再灌注损伤'+'大脑中动脉阻塞'+'大脑中动脉闭塞'+'MCAO'+'MCAO/R'+'I/R'+'CIRI')  AND  SU=('肠道菌群'+'肠道微生物'+'肠道微生态'+'肠道菌群失调'+'肠-脑轴'+'脑-肠轴'+'菌群-肠-脑轴'+'脑肠轴'+'肠脑轴'+'短链脂肪酸'+'SCFAs'+'肠屏障'+'肠黏膜屏障'+'肠通透性'+'肠道通透性'+'紧密连接'+'闭锁小带蛋白-1'+'ZO-1'+'Occludin'+'Claudin'+'内毒素'+'脂多糖'+'LPS'+'DAO'+'D-乳酸'+'氧化三甲胺'+'TMAO'+'吲哚-3-丙酸'+'IPA')  AND  SU=('动物'+'大鼠'+'小鼠'+'SD大鼠'+'Wistar大鼠'+'Sprague-Dawley大鼠'+'C57BL/6小鼠') | 36 |

**VIP**

| # | searches | resultes |
| --- | --- | --- |
| 1 | M=(电针 OR 电针疗法 OR 电针预处理 OR 电针治疗)  AND  M=(缺血性脑卒中 OR 缺血性卒中 OR 脑缺血 OR 脑梗死 OR 脑梗塞 OR 脑缺血再灌注 OR 脑缺血再灌注损伤 OR 大脑中动脉闭塞 OR MCAO)  AND  M=(肠道菌群 OR 肠道微生物 OR 肠道微生态 OR 肠道菌群失调 OR 脑肠轴 OR 肠脑轴 OR 肠屏障 OR 肠黏膜屏障 OR 肠通透性 OR 脂多糖 OR 内毒素 OR DAO OR D-乳酸 OR 短链脂肪酸)  AND  M=(大鼠 OR 小鼠 OR SD大鼠 OR Wistar大鼠 OR Sprague-Dawley大鼠 OR C57BL/6小鼠) | 15 |

**Wangfang**

| # | searches | resultes |
| --- | --- | --- |
| 1 | 主题:(电针 OR 电针疗法 OR 电针预处理 OR 电针治疗)  AND  主题:(缺血性脑卒中 OR 缺血性卒中 OR 脑缺血 OR 脑梗死 OR 脑梗塞 OR 脑缺血再灌注 OR 脑缺血再灌注损伤 OR 大脑中动脉阻塞 OR 大脑中动脉闭塞 OR MCAO OR MCAO/R OR I/R OR CIRI)  AND  主题:(肠道菌群 OR 肠道微生物 OR 肠道微生态 OR 肠道菌群失调 OR 肠-脑轴 OR 脑-肠轴 OR 菌群-肠-脑轴 OR 短链脂肪酸 OR SCFAs OR 肠屏障 OR 肠黏膜屏障 OR 肠通透性 OR 肠道通透性 OR 紧密连接 OR 闭锁小带蛋白-1 OR ZO-1 OR Occludin OR Claudin OR 内毒素 OR 脂多糖 OR LPS OR DAO OR D-乳酸 OR 氧化三甲胺 OR TMAO OR 吲哚-3-丙酸 OR IPA)  AND  主题:(动物 OR 大鼠 OR 小鼠 OR SD大鼠 OR Wistar大鼠 OR Sprague-Dawley大鼠 OR C57BL/6小鼠) | 54 |

**CBM**

| # | searches | resultes |
| --- | --- | --- |
| 1 | 电针 OR 电针疗法 OR 电针预处理 OR 电针治疗 | 39,640 |
| 2 | 缺血性脑卒中 OR 缺血性卒中 OR 脑缺血 OR 脑梗死 OR 脑梗塞 OR 脑缺血再灌注 OR 脑缺血再灌注损伤 OR 大脑中动脉阻塞 OR 大脑中动脉闭塞 OR MCAO OR MCAO/R OR I/R OR CIRI | 497,730 |
| 3 | 肠道菌群 OR 肠道微生物 OR 肠道微生态 OR 肠道菌群失调 OR 肠-脑轴 OR 脑-肠轴 OR 菌群-肠-脑轴 OR 短链脂肪酸 OR SCFAs OR 肠屏障 OR 肠黏膜屏障 OR 肠通透性 OR 肠道通透性 OR 紧密连接 OR 闭锁小带蛋白-1 OR ZO-1 OR Occludin OR Claudin OR 内毒素 OR 脂多糖 OR LPS OR DAO OR D-乳酸 OR 氧化三甲胺 OR TMAO OR 吲哚-3-丙酸 OR IPA | 543,799 |
| 4 | 动物 OR 大鼠 OR 小鼠 OR SD大鼠 OR Wistar大鼠 OR Sprague-Dawley大鼠 OR C57BL/6小鼠 | 9,433,555 |
| 5 | #1 AND #2 AND #3 AND #4 | 24 |
